# Revealing subterahertz atomic vibrations in quantum paraelectrics by surface-sensitive spintronic terahertz spectroscopy

**DOI:** 10.1126/sciadv.ads8601

**Published:** 2024-11-29

**Authors:** Zhaodong Chu, Junyi Yang, Yan Li, Kyle Hwangbo, Jianguo Wen, Ashley R. Bielinski, Qi Zhang, Alex B. F. Martinson, Stephan O. Hruszkewycz, Dillon D. Fong, Xiaodong Xu, Michael R. Norman, Anand Bhattacharya, Haidan Wen

**Affiliations:** ^1^ Materials Science Division, Argonne National Laboratory, Lemont, IL 60439, USA.; ^2^ Department of Physics, University of Washington, Seattle, WA 98195, USA.; ^3^ Center for Nanoscale Materials, Argonne National Laboratory, Lemont, IL 60439, USA.; ^4^ Advanced Photon Source, Argonne National Laboratory, Lemont, IL 60439, USA.

## Abstract

Understanding surface collective dynamics in quantum materials is crucial for advancing quantum technologies. For example, surface phonon modes in quantum paraelectrics are thought to be essential in facilitating interfacial superconductivity. However, detecting these modes, especially below 1 terahertz, is challenging because of limited sampling volumes and the need for high spectroscopic resolution. Here, we report surface soft transverse optical (TO1) phonon dynamics in KTaO_3_ and SrTiO_3_ by surface-sensitive spintronic terahertz spectroscopy that can sense the collective modes only a few nanometers deep from the surface. In KTaO_3_, the TO1 mode softens and sharpens with decreasing temperature, leveling off at 0.7 terahertz. In contrast, this mode in SrTiO_3_ broadens substantially below the quantum paraelectric crossover and coincides with the hardening of a sub–milli–electron volt phonon mode related to the antiferrodistortive transition. These observations that deviate from their bulk properties may have implications for interfacial superconductivity and ferroelectricity. The developed technique opens opportunities for sensing low-energy surface collective excitations.

## Teaser

Ultrafast terahertz emission captures surface soft phonon modes that deviate from their bulk properties in quantum paraelectrics.

## INTRODUCTION

Surface phonons—collective vibrations of atoms at an interface or surface that span only a few nanometers along the depth direction—are pivotal to surface dynamics and properties. Their unique characteristics can deviate substantially from the bulk and have been found to underlie a plethora of exotic nanoscale phenomena such as surface phonon polaritons (*1*), interfacial superconductivity (*2*–*5*), surface catalysis (*6*), and interfacial thermal transport (*7*). Understanding and controlling these collective dynamics at the surface are essential for applications. For instance, in quantum paraelectrics such as KTaO_3_ (KTO) and SrTiO_3_ (STO), recent studies (*3*–*5*) suggest that the observed interfacial superconductivity is driven by the coupling of the electrons to the low-energy surface transverse optical (TO1) phonon. Variations in the superconducting transition temperature with interface orientation in these quantum paraelectrics were proposed to result from the distinct surface TO1 mode dynamics unique to each material (*4*, *5*). However, direct experimental evidence is challenging to obtain because of the lack of experimental techniques capable of probing low-energy surface phonon modes (<1 THz) with the required spectroscopic resolution and limited sampling volumes.

Conventional tools such as Raman (*8*), hyper Raman (*9*), and terahertz (THz) spectroscopy (*10*) rely on far-field light-matter interactions with the optical penetration depth typically on the order of tens to thousands of nanometers and are thus not sensitive to surface modes. The advances in tip-enhanced photonic techniques, such as THz scanning near-field optical microscope (*11*, *12*), provide near-field light-matter interactions. However, because of the long wavelength of the THz radiation (~300 μm), the minimum probing depth can only approach tens of nanometers. Similar challenges remain for tip-enhanced Raman spectroscopy (*13*), where it is difficult to separate the bulk contribution from the surface one. Planar tunneling into conducting STO, though sensitive well below the milli–electron volt level, has not been able to detect the coupling of electrons to the low-energy transverse optical phonons (*14*, *15*). Other scattering spectroscopies such as laser-induced scanning tunneling microscopy (*16*), surface-enhanced Raman scattering (*17*), electron energy-loss spectroscopy (*18*, *19*), and helium atom scattering (*20*) can achieve the needed surface sensitivity, but their energy resolution is limited to tens of milli–electron volts. On the other hand, neutron scattering (*21*) can achieve sub–milli–electron volt energy resolution, but it falls short in measuring surface modes because of the substantial sample volume required for measurements. In this context, sensitive probes of low-energy collective dynamics that are confined within a few nanometers from a bulk crystal surface with milli–electron volt energy sensitivity are urgently needed.

Here, we introduce a surface-sensitive spintronic THz spectroscopy (SSTS) where both the cavity-enhanced near-field THz-matter interaction and ultrafast coupling between the interfacial transient current and the sample are confined to the nanoscale in the vicinity of the sample surface for detecting ultralow-energy surface modes. The probing depth of SSTS in KTO and STO was found to be ~5 nm. Our approach differs from traditional THz surface spectroscopy (*22*–*25*), where the absorption depth of the far-field optical pump light defines a probe depth typically on the order of tens to hundreds of nanometers. Although this depth can be regarded as a surface probe in a certain context (*25*), it is much deeper than the few nanometers near an interface where exotic quantum processes occur such as interfacial superconductivity in quantum paraelectrics (*2*–*5*). Using SSTS, we uncovered the distinct dynamics of surface milli–electron volt phonons that have thus far been elusive in two representative quantum paraelectric crystals, KTO and STO. Our temperature-dependent phonon spectra revealed an unexpected broadening of the TO1 mode at the STO surface, in contrast with the sharp TO1 mode found in bulk STO as well as at the KTO surface. These observations potentially shed light on the origin of differing interfacial superconducting transitions among quantum paraelectrics, as the surface TO1 mode is thought to play an important role in the Cooper pairing process. Our work showcases SSTS’s capability to provide unprecedented insights into surface and interfacial dynamics in quantum materials.

## RESULTS

### Surface-sensitive spintronic THz spectroscopy

**Fig. 1.**
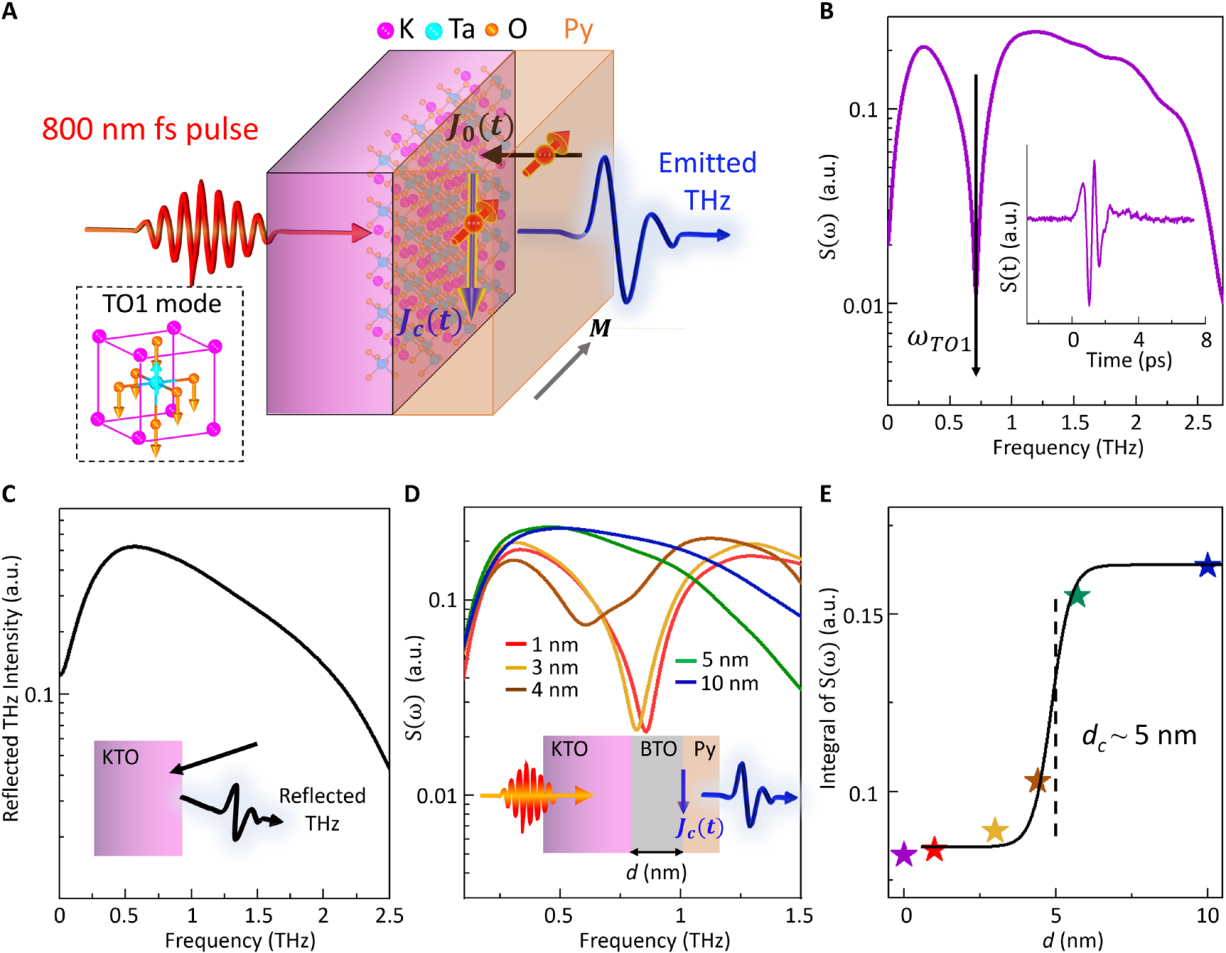
Surface-sensitive spintronic THz spectroscopy. (**A**) Schematic representation of the THz emission in SSTS, using a Py/KTO (111) sample as an example. Laser-excited spin-polarized hot electrons [i.e., $J_{0}\left(t\right)$] diffuse toward the Py-KTO interface and subsequently transform into a charge current $J_{c}\left(t\right)$ through spin-charge conversion. $J_{c}\left(t\right)$ is the source of the THz radiation. “M” and the gray arrow represent the in-plane magnetization of Py. The black dashed rectangle shows a diagram of the TO1 phonon mode in a KTO unit cell. (**B**) THz emission spectrum from Py/KTO (111), where the dip indicates the TO1 mode frequency $\omega_{\text{TO}1}$. Inset: Corresponding THz time-domain waveform. (**C**) Reflected THz spectrum at the KTO surface, where no TO1 phonon dip is observed. The inset shows a simple schematic of the THz reflection measurement (see fig. S6 for more details). (**D**) THz emission from Py/BTO-buffer/KTO (111) samples with various buffer thicknesses. The inset shows a schematic of the sample structures with a BTO-buffer layer between the KTO surface and the Py layer, where *d* denotes the thickness of the buffer layer. (**E**) Integral of the THz spectra versus the thickness of the spacer. A tanh function fitting $\int S\left(\omega \right)d\omega =a+b\;\text{tanh}\;\left(\frac{d-d_{c}}{c}\right)$ indicates that the probe depth *d*_c_ of SSTS is about 5 nm, as marked by the dashed black line. The purple star in (E) presents the result with no buffer layer as shown in (B). All the results shown in this figure were taken at *T* = 10 K, and the THz field was detected on the Py side.

The design of SSTS is based on THz spintronic emission via spin-to-charge current conversion, where a transient current (i.e., the THz source) is confined within a nanometer-thin metallic layer or interface. Previous THz spintronics studies based on metal/metal (*26*–*32*) and metal/insulator (*33*–*38*) heterostructures primarily focused on optimizing efficiency and bandwidth as an intense THz source and exploring various spin-to-charge conversion mechanisms. This work illustrates that SSTS based on dielectric-ferromagnetic metal heterostructures can probe the collective dynamics confined within a few nanometers of the underlying substrate’s surface. As exemplified by a Py/KTO (111) heterostructure shown in Fig. 1A, a 3-nm-thick, in-plane magnetized ferromagnet (FM) layer, permalloy (Py: Ni_0.8_Fe_0.2_), was deposited on the single crystal sample, with atomically sharp interfaces (Materials and Methods and fig. S1). An 800-nm, 100-fs laser pulse is passed through the transparent sample, exciting the Py layer (Materials and Methods). The resulting spin superdiffusion current $J_{0}\left(t\right)$ (the black arrow in Fig. 1A) is converted into an in-plane, spin-polarized charge current $J_{c}\left(t\right)$ via spin-to-charge conversion at the interface (*36*–*39*) (the downward-pointing blue arrow in Fig. 1A). This interfacial transient current $J_{c}\left(t\right)$ serves as the source of the THz radiation, and the resulting far-field radiation S(t) is detected by free-space, electro-optic sampling (Materials and Methods and fig. S2). To verify the spintronic origin of the THz generation via spin-to-charge conversion, rather than the photo-Dember effect (*22*, *23*) or the surface surge currents (*24*) reported in semiconductors, we measured the expected dependence of the THz polarization on the magnetization direction in the Py/KTO and Py/STO samples, and we also found no THz emission from either bare KTO (or STO) crystals or nonmagnetic metal (NM)/STO heterostructures under the same laser excitation conditions (fig. S3). Furthermore, we confirmed that the contribution of ultrafast demagnetization-induced magnetic dipole radiation in our samples is negligible (fig. S4).

Figure 1B displays the measured THz waveform of the Py/KTO (111) sample and its Fourier spectrum at the temperature *T* = 10 K. A prominent spectral dip at the TO1 phonon mode frequency of the sample is evident in the emitted THz spectrum, in contrast to the broadband THz emission in a sapphire reference sample that is featureless in the measured THz window (fig. S5). We demonstrated that the TO1 spectral dip is not due to the THz absorption by the KTO substrate by carrying out far-field THz reflection measurements (detailed in fig. S6). Figure 1C shows the reflected THz spectrum from a KTO (111) substrate at *T* = 10 K, where no spectral dip is observed. Moreover, we found that spatially separating the THz source $\left[J_{c}\left(t\right)\right]$ from the sample by adding THz featureless spacers (fig. S7), such as 100-nm-thick SiO_2_ or a few micrometer-thick air gap, between the sample and the Py layer, prevents the detection of the TO1 spectral dip in the emitted THz radiation, although the THz field in the Py layer can still pass through the spacer layer to probe KTO and the reflected THz field was recorded. These results confirm that the absorption of far-field THz radiation is not the mechanism of SSTS. Our theoretical modeling of SSTS suggests that the detection mechanism has two contributors. One is cavity-enhanced near-field THz-matter interaction at the interface, where the 3-nm Py layer acts as an ultra-thin Fabry-Pérot cavity, allowing the near-field THz to directly sense the cavity impedance and the dielectric response of the interfaces. The other is the polarization modulation at the KTO surface, induced by the interfacial current $J_{c}\left(t\right)$ because of hot electron–TO1 phonon coupling, which interferes destructively with $J_{c}\left(t\right)$. As detailed in the “Modeling SSTS” section later in the main text, the two contributions enable the surface TO1 mode to be detected as a spectral dip in the emitted THz field.

To experimentally quantify the probe depth of the surface TO1 mode in KTO and establish the interface sensitivity of SSTS, we carried out experiments in which the thickness of a buffer layer between the Py and the KTO substrate was varied. We chose BaTiO_3_ (BTO), another member of the perovskite oxide family, as the buffer layer, which has a similar electrical bandgap and high dielectric constant as the substrate but does not host any modes within our THz detection window (*40*). The BTO-buffer layer was deposited by pulsed laser deposition (see Materials and Methods) to achieve atomically sharp interfaces with nanometer thickness (fig. S8) so that the leakage current was eliminated. Figure 1D presents the emitted THz spectrum S(ω) from Py/BTO-buffer/KTO (111) samples at *T* = 10 K as a function of the buffer layer thickness. The spectral dip positions vary as the buffer layer thickness increases from 1 to 4.4 nm, suggesting that the spectral dip does not arise from a bulk phonon mode as the substrate is the same for these samples. When the thickness of the buffer layer is increased beyond 5.7 nm, no spectral dip is observed in the detection window. This result demonstrates that a BTO-buffer layer with a thickness larger than 5.7 nm effectively prevents the detection of the KTO surface TO1 phonon. We quantified the critical BTO-buffer layer thickness (*d_c_*) to be ~5 nm as the probe depth for detecting the KTO TO1 mode, as shown in Fig. 1E. Such a probe depth matches the width of the two-dimensional electron gas (2DEG) formed at KTO or STO interfaces/surfaces (*41*). Therefore, SSTS is a powerful tool for revealing the dynamics of the surface TO1 phonons involved in the unconventional superconductivity at quantum paraelectric interfaces. In addition, SSTS does not require a strong nonlinear optical response at the interface as needed for the differential frequency generation process (*42*).

### Surface phonon dynamics of KTO and STO

**Fig. 2.**
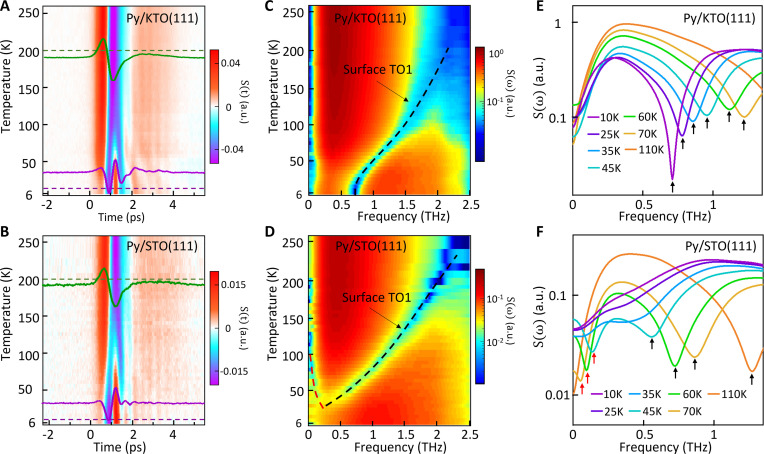
Visualization of sub-THz phonons at KTO (111) and STO (111) surfaces. (**A** and **B**) Images of the temperature-dependent THz waveforms of Py/KTO (111) and Py/STO (111) samples, respectively. (**C** and **D**) Fourier spectra of (A) and (B), respectively. The black dashed curves in (C) and (D) mark the softening of the surface TO1 mode of KTO and STO, respectively. The red dashed curve in (D) highlights the hardening of the mode related to antiferrodistortion at the STO surface. (**E** and **F**) Line-cut THz spectral profiles at selected temperatures of KTO and STO samples, respectively. (E) illustrates the sharpening of the KTO surface TO1 mode spectral dip (marked by black arrows) as the temperature decreases, and (F) shows the broadening and shallowing of the STO surface TO1 mode spectral dip (marked by the black arrows) and the sub–milli–electron volt mode spectral dip (marked by red arrows) at low temperatures.

By applying SSTS, we uncovered the dynamics of surface milli–electron volt phonon modes for two representative quantum paraelectrics, KTO and STO. Although the temperature-dependent evolution of the THz waveforms in both samples exhibited similarities at high temperatures (as indicated by the green curves in the time trace at 200 K in Fig. 2, A and B), distinct differences emerged in the low-temperature regime, as highlighted by the time traces at 10 K (purple curves in Fig. 2, A and B). The evolution of the THz spectra was primarily driven by the temperature-dependent surface TO1 phonon dynamics (Fig. 2, C and D). At temperatures above 200 K, the spectral dips were less pronounced because of the limited THz detection window. At low temperatures, distinct spectral features from surface modes were observed. For KTO (Fig. 2, C and E), the dip corresponding to the TO1 mode sharpened as the temperature decreased, consistent with reduced thermal fluctuations. Moreover, the softening of the mode ceased below ~15 K, leveling off at a mode frequency of 0.7 ± 0.02 THz (the error bar of the mode frequency is determined in fig. S9). This observation was reliably reproduced in measurements on two other Py/KTO (111) samples prepared in the same way, as shown in Fig. 1B and fig. S10. The THz spectra of the Py/STO (111) sample (Fig. 2, D and F) exhibited characteristics at low temperatures that differ from what was observed in Py/KTO (111). In the STO sample, the surface TO1 mode dip was shallower and broader at temperatures below ~60 K compared to KTO and eventually became featureless below ~35 K, the crossover temperature between paraelectric and quantum paraelectric behavior (*43*, *44*). Moreover, a sub–milli–electron volt mode, highlighted by the red dashed curve in Fig. 2D and red arrows in Fig. 2F, emerged below ~100 K and then hardened as the temperature was further lowered. The spectroscopic dip for this lower-energy mode, similar to the TO1 mode, broadened as the temperature decreased and was not discernable at temperatures below ~35 K. The emergence of this surface sub–milli–electron volt (<0.25 THz) mode below 100 K is likely associated with the antiferrodistortive transition from the cubic to the tetragonal phase (*45*–*47*). In bulk STO, the antiferrodistortive transition occurs at 105 K, related to phonon softening at the *R* point of the Brillouin zone. Below this transition, the unit cell doubles, allowing the *R* point phonon to manifest at the Γ point. Although this phonon has even parity, inversion symmetry breaking at the interface can render it infrared (IR) active, leading us to conjecture that this is the origin of the emergent sub–milli–electron volt mode. As expected, this phonon hardens below the antiferrodistortive transition. In bulk STO, two phonons appear below 105 K as the triply degenerate *R* phonon splits into a doubly degenerate *E* mode and a singly degenerate *A* mode due to the symmetry reduction from cubic to tetragonal. The lower-energy *E* mode at low temperatures is ~0.45 THz in crystals (*46*) and 1.2 THz in polycrystalline ceramics (*47*), both much higher than the sub–milli–electron volt mode we observed. This suggests that the *R*-point phonon may be softer at the surface than in the bulk or that the splitting of the *A* and *E* modes is more pronounced at the surface or interface [note that the *A* mode is not IR active (*47*)]. In addition, the two broad surface phonons (i.e., the sub–milli–electron volt mode and the TO1 mode) merge at low temperatures (Fig. 2, D and F), suggesting an interaction between the two modes that is not evident in STO bulk samples. Thus, our observations show a strong deviation between bulk STO and the surface.

**Fig. 3.**
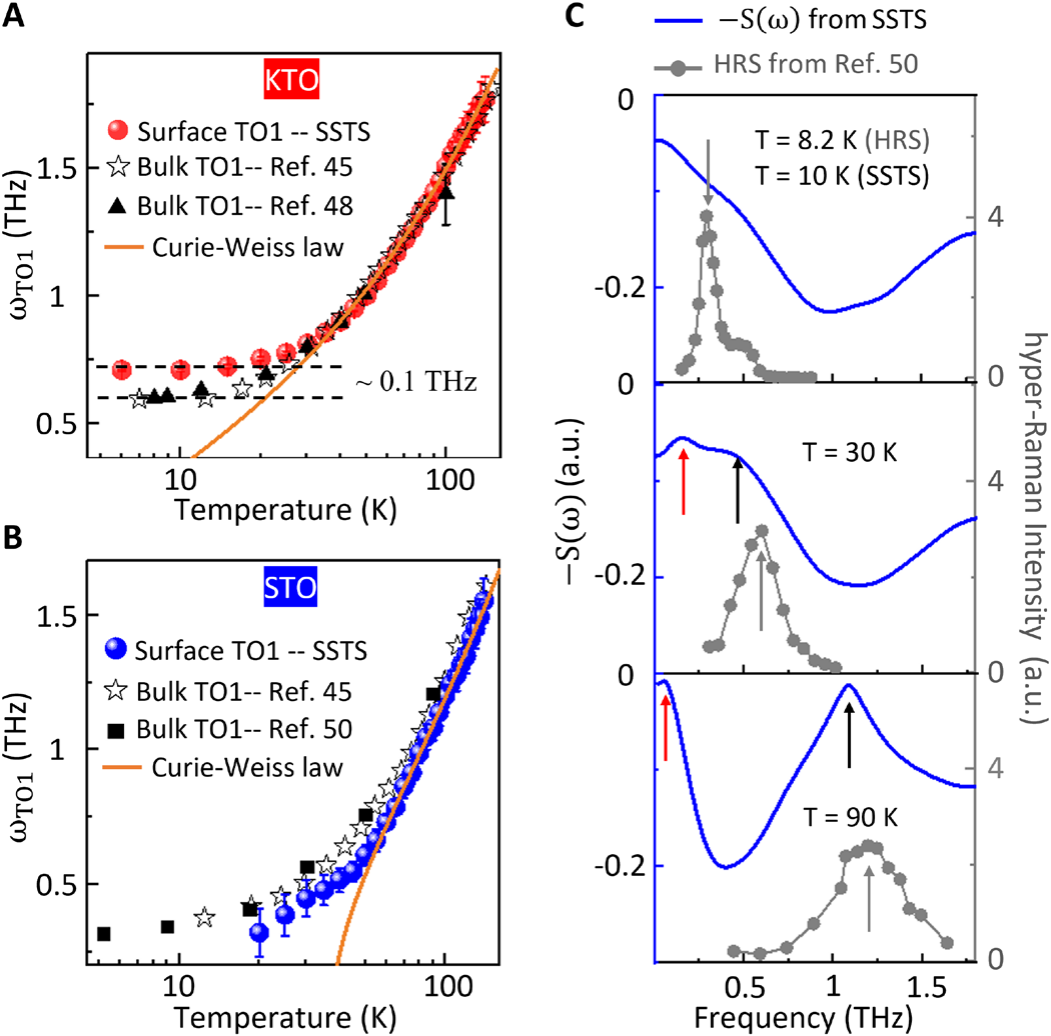
Temperature-dependent phonon frequencies in KTO and STO samples. (**A**) Comparison of the KTO surface TO1 and bulk TO1 mode frequencies. The data from THz transmission spectroscopy and hyper-Raman spectroscopy (HRS) are adapted from (*48*) and (*45*), respectively. (**B**) Comparison of STO surface TO1 and bulk TO1 mode frequencies. In SSTS measurements, the surface TO1 mode frequencies below 20 K could not be determined given the broadness of the spectral dip and its overlap with the sub–milli–electron volt mode. The data of HRS are adapted from (*45*, *50*). The orange curves in (A) and (B) represent the Curie-Weiss law fitting $\omega_{\text{TO}1}\propto \sqrt{T-T_{c}}$ with $T_{c}$ ~ 5 K in (A) and ~ 37.5 K in (B), respectively. (**C**) Comparison of spectral sharpness between surface TO1 and bulk TO1 modes in STO. The HRS measurements [adapted from (*50*)] show a sharpening of the bulk TO1 mode in an STO crystal as the temperature decreases. The blue curves present plots of the reversed SSTS spectral data [i.e., −S(ω)] of the Py/STO (111) sample from Fig. 2D, providing a direct comparison of spectroscopic peak-to-peak characteristics with (*50*). At lower temperatures, the SSTS spectra do not exhibit pronounced peaks, because of the broadening of the surface TO1 mode. Here, the black (SSTS) and gray (HRS) arrows mark the TO1 mode positions, and the red (SSTS) arrows mark the surface sub–milli–electron volt mode.

To further compare the TO1 mode at the surface and in the bulk of both KTO and STO, we conducted the following analysis. Figure 3A illustrates the frequencies of the surface and bulk TO1 modes in KTO as a function of temperature. Notably, above 30 K, the mode frequencies of the surface and bulk were approximately the same, following a temperature dependence that is consistent with the Curie-Weiss law (i.e., $\omega_{\text{TO}1}\propto \sqrt{T-T_{c}}$). Below 30 K, the softening of both the surface and bulk modes was gradually suppressed, deviating from the Curie-Weiss law at the paraelectric to quantum paraelectric crossover temperature. The surface TO1 frequency was found to be ~0.1 THz higher than that of the bulk KTO mode reported in the literature (*45*, *48*), likely because of the presence of a static internal electric field at the Py/KTO interface (*49*).

A comparison of the TO1 mode at the STO surface and the bulk is shown in Fig. 3 (B and C). Above 60 K, the TO1 mode frequency for both surface and bulk aligned with the Curie-Weiss law, with a fitted transition temperature *T*_c_ of 37 K. Below 60 K, the softening deviated from the Curie-Weiss law, as expected. However, unlike KTO, the STO surface phonon appears to be softer than the bulk one. Similar broadening of the THz spectroscopic dip from the surface TO1 phonon was also observed for a Py/STO (001) sample in the same temperature range (fig. S10). The broadening of the surface phonon linewidth observed by SSTS at low temperatures has not been previously reported in STO crystals (*45*, *47*, *50*) or films (*49*, *51*). Prior hyper-Raman spectroscopy measurements (*50*) (Fig. 3C) showed that the bulk TO1 mode linewidth sharpens as the temperature decreases, opposite to what we observed at the surface with SSTS. This sharpening of the bulk linewidth was corroborated for the *A_1g_* and *E_g_* phonons measured by Raman spectroscopy on the same Py/STO (001) sample in this study (fig. S11).

The substantial broadening of the surface TO1 mode below the quantum paraelectric crossover indicates strong fluctuations of the TO1 modes at the STO surface. These fluctuations may affect how the surface TO1 mode mediates Cooper pairing and therefore the interfacial superconducting transition temperatures. For example, the transition temperature for superconductivity of the 2DEG at the STO (111) interface is in the range of hundreds of millikelvin, an order of magnitude lower than that for the 2DEG at the KTO (111) interface (*3*, *4*). Similarly, the surface TO1 spectral dips of a KTO (001) 2DEG sample, where the 2DEG remains normal down to tens of millikelvin (*3*, *4*), are also much broader than those of the KTO (111) 2DEG (fig. S12). The fluctuation being more pronounced in STO than in KTO could be due to STO’s closer proximity to the ferroelectric quantum critical point (*52*), which makes the behavior of STO more dependent on its surface or interface reconstruction and imperfections (*53*–*55*), leading to spectral broadening of the surface TO1 mode. Although these results alone would not explain why doped bulk STO and STO (111) interfaces exhibit similar superconducting transition temperatures (*56*, *57*), our findings offer valuable insights into the interfacial superconductivity of quantum paraelectrics. In addition, they underscore the exceptional capabilities of SSTS in revealing low-energy interfacial structural dynamics.

### Modeling SSTS

As shown in our experiments (Fig. 1, C and D, and figs. S6 and S7), the underlying mechanism of SSTS in detecting surface modes is not due to the absorption of the THz waves because the reflected THz wave at the far field does not encode the phonon information. To understand the underlying detection mechanism of SSTS, we model the cavity-enhanced near-field THz-matter interactions and electron-phonon couplings as detailed below.

The purpose of depositing a 3-nm-thick Py layer on the sample surface is twofold: It serves as the source for spintronic THz generation as well as provides a Fabry-Pérot cavity. This cavity confines the generated THz field, thereby enhancing near-field THz-matter interactions at the interface. The generated near-field THz $E_{\text{near}}\left(\omega \right)$ inside the cavity can be approximately expressed as a generalized Ohm’s law (*26*, *27*)

$$E_{\text{near}}\left(\omega \right)=Z\left(\omega \right)J_{c}\left(\omega \right)=\frac{Z_{0}}{n_{\text{air}}\left(\omega \right)+n_{\text{samp}}\left(\omega \right)+Z_{0}G}J_{c}\left(\omega \right)$$ (1)

**Fig. 4.**
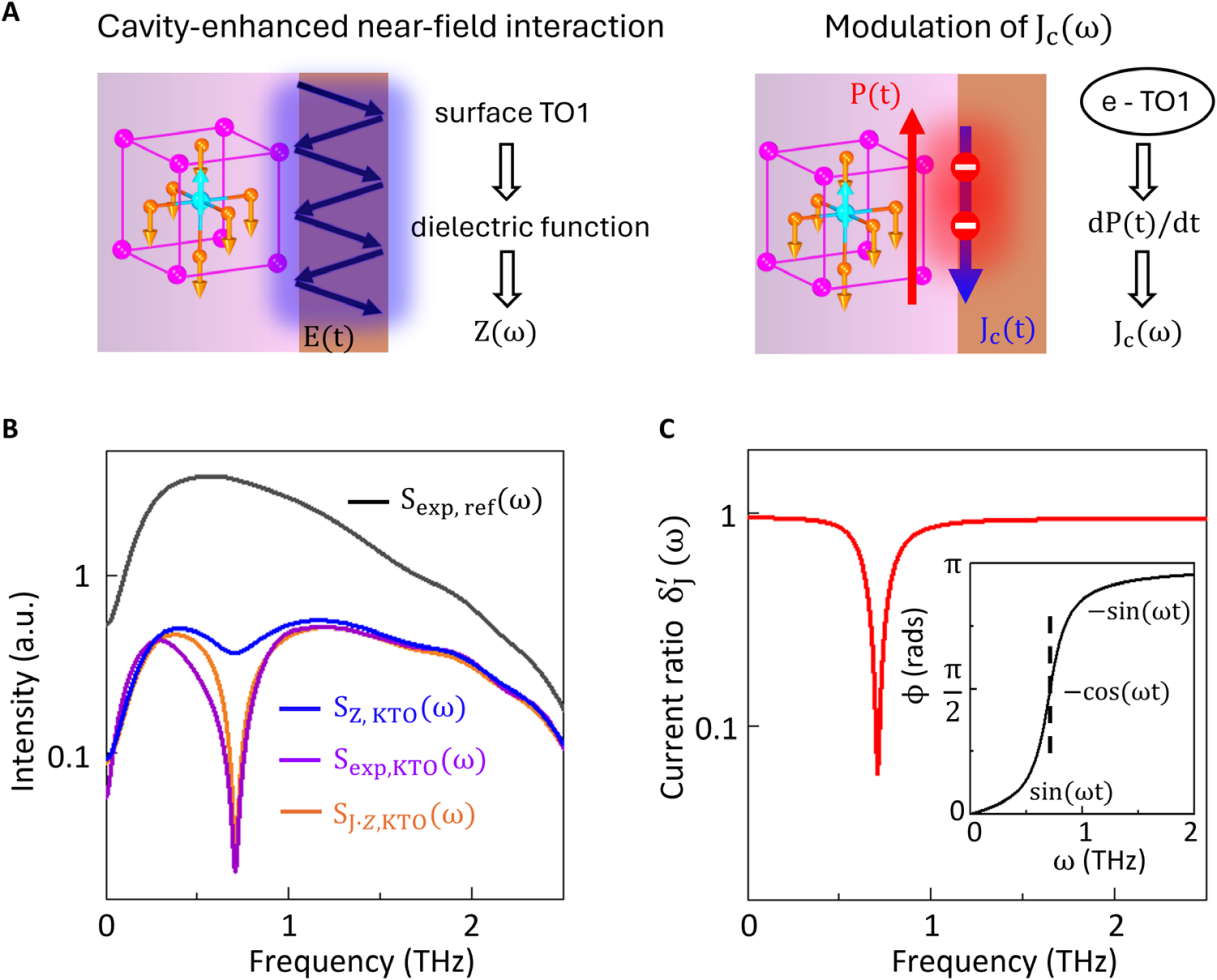
Theoretical modeling. (**A**) Schematic of the detection mechanisms of SSTS. Left: Detection of surface modes through near-field interactions. The Py layer functions as a thin cavity where all reflected THz fields (black arrows) within the cavity constructively interfere, facilitating near-field THz-matter interaction. The blue glow of the THz field illustrates the depth of the near-field THz-matter interaction. The effective near-field THz within the cavity is represented by E(t) with its Fourier spectrum denoted as $E_{\text{near}}\left(\omega \right)$. Z(ω) is the cavity impedance, encapsulating the spectral information of the surface TO1 mode through the dielectric function. Right: Detection of surface modes via modulation of the surface current, $J_{c}\left(\omega \right)$. The spin-polarized electrons (red balls) involved in the transient current $J_{c}\left(t\right)$ (blue arrow) couple with the surface TO1 mode, so that $J_{c}\left(t\right)$ can induce a surface in-plane transient polarization P(t) with its derivative dP(t)/dt representing a transient current that destructively interferes with $J_{c}\left(t\right)$. The red glow indicates the tunneling depth of the hot electrons that determines the probing depth of $J_{c}\left(\omega \right)$. (**B**) Comparison between the experimental data $S_{\text{exp},\text{KTO}}\left(\omega \right)$ and $S_{\text{exp},\text{ref}}\left(\omega \right)$ at 6 K, with calculated THz spectra: $S_{Z,\text{KTO}}\left(\omega \right)$ including the dielectric contribution alone; $S_{\mathrm{JZ},\text{KTO}}\left(\omega \right)$ including the contribution from both the dielectric response and the induced *dP*/*dt* term. (**C**) Calculated $\delta_{J}^{\prime }\left(\omega \right)$ profile with $c_{\text{ind}}$= 1.63 × 10^−4^, $\gamma_{\text{TO}1}$ = 0.24 THz used in (B). The inset shows the phase angle (ϕ) of the dielectric function as a function of frequency ω. At $\omega_{\text{TO}1}$ (the black dashed line), $\phi =\frac{\pi }{2}$, the induced current contribution ∝−cos(ωt) and thus destructively interferes with the driving current $J_{c}\left(t\right)$ ∝cos(ωt).

Here, $E_{\text{near}}\left(\omega \right)$ and $J_{c}\left(\omega \right)$ are the Fourier transforms of E(t) and $J_{c}\left(t\right)$, respectively. Z(ω) is the impedance of the cavity, $Z_{0}$ is the vacuum impedance, and *G* is the conductance of the Py film. $n_{\text{air}}$ and $n_{\text{samp}}$ are the complex refractive indices of air and the sample (i.e., KTO or STO), respectively. The measured THz spectrum S(ω) is the far-field radiation that is convolved with the propagation of $E_{\text{near}}\left(\omega \right)$ from the Py to the detector and the detector response function H(ω), i.e., $S\left(\omega \right)=E_{\text{near}}\left(\omega \right)H\left(\omega \right)$. From Eq. 1, there are two contributions to the detection of the surface modes such as the TO1 mode. As depicted in Fig. 4A, one is the impedance of the cavity, *Z*(ω), which characterizes the near-field THz-matter interaction at the interface via its dielectric response as the spectral information of the surface TO1 mode is encoded in the dielectric function. The other is the transient current source term, $J_{c}\left(\omega \right)$, which can be modulated by the surface polarization, dP(t)/dt, induced by hot electron–TO1 phonon interactions. We quantitatively examine the contribution of the two terms separately, as described below.

We first focus on the contribution of the impedance term *Z*(ω) by making the approximation that $J_{c}\left(\omega \right)$ has no frequency structure because of the surface modes and thus assume that the ratio of $J_{c}\left(\omega \right)$ for the sample (Py/KTO or Py/STO) to that of the reference (Py/sapphire) is a constant, i.e., $\delta_{J}=\frac{J_{c,\text{samp}}\left(\omega \right)\;}{J_{c,\text{ref}}\left(\omega \right)}=C$. By dividing the experimental THz spectrum for the sample [$S_{\text{exp},\text{samp}}\left(\omega \right)$] and the reference $\left[S_{\text{exp},\text{ref}}\left(\omega \right)\right]$, one obtains

$$\frac{S_{\text{exp},\text{samp}}\left(\omega \right)}{S_{\text{exp},\text{ref}}\left(\omega \right)}=\frac{J_{c,\text{samp}}\left(\omega \right)\left|1+n_{\text{ref}}+Z_{0}G\right|}{J_{c,\text{ref}}\left(\omega \right)\left|1+n_{\text{samp}}\left(\omega \right)+Z_{0}G\right|}=C\frac{\left|1+n_{\text{ref}}+Z_{0}G\right|}{\left|1+n_{\text{samp}}\left(\omega \right)+Z_{0}G\right|}$$ (2)

This division has the additional benefit of removing H(ω). On the basis of Eq. 2, the THz signal from KTO or STO is expected to be suppressed relative to the reference because the refraction index of the KTO or STO ($n_{\text{samp}}$) is much larger than that of sapphire ($n_{\text{ref}}$ = 3.31) and *Z*_0_*G* (estimated to be 1.7; see fig. S13 caption), in agreement with our data (fig. S13). Assuming Eq. 2 is valid, the index of refraction of the sample surface can be determined solely from the measured THz spectra (fig. S13). We can also use Eq. 2 to estimate the THz spectrum at 6 K for KTO from the contribution of *Z*(ω) alone from $S_{Z,\text{KTO}}\left(\omega \right)=C\frac{S_{\text{exp},\text{ref}}\left(\omega \right)\left|1+n_{\text{ref}}+Z_{0}G\right|}{\left|1+n_{\text{KTO}}\left(\omega \right)+Z_{0}G\right|}$ where $S_{\text{exp},\text{ref}}\left(\omega \right)$ is the experimental data of Py/sapphire at 6 K. $n_{\text{KTO}}$ is modeled using the Lyddane-Sachs-Teller relation (*58*), ${n^{2}}_{\text{KTO}}\left(\omega \right)=\varepsilon^{*}\left(\omega \right)=\varepsilon \left(\infty \right)\prod_{i}^{n}\frac{\omega_{\text{LOi}}^{2}-\omega^{2}+i\omega \gamma_{\text{LOi}}}{\omega_{\text{TOi}}^{2}-\omega^{2}+i\omega \gamma_{\text{TOi}}}$, where the frequencies of the various longitudinal ($\omega_{\text{LOi}}$) and transverse ($\omega_{\text{TOi}}$) optical phonon modes are from IR measurements (*58*). To make a more robust comparison to our experimental data $S_{\text{exp},\text{KTO}}\left(\omega \right)$ at 6 K, we adjusted the TO1 mode frequency from the IR value of 0.6 to 0.71 THz to match the location of the spectral dip, and we fixed *C* (1.62) by matching to the data at the upper range of our THz window.

The probing depth of the cavity impedance *Z*(ω) based on the dielectric response is determined by the depth of the near-field enhanced region close to the cavity. This depth can be as short as a few nanometers in materials with a large dielectric constant, as we experimentally demonstrated using a BTO buffer layer (Fig. 1D). To assess this depth for low dielectric materials, we replaced the BTO buffer layer with Al_2_O_3_ and found that the probe depth of *Z*(ω) in Al_2_O_3_ extends from tens of nanometers to a hundred nanometers (fig. S14). Therefore, the strong resonance of the TO1 mode in our THz window that gives rise to a large dielectric response is important to shorten the probe depth of the *Z*(ω) term.

Figure 4B shows the comparison of spectral profiles between $S_{Z,\text{KTO}}$ (ω) and experimental results at 6 K, i.e., $S_{\text{exp},\text{KTO}}\left(\omega \right)$ and $S_{\text{exp},\text{ref}}\left(\omega \right)$. Although the spectrum of $S_{Z,\text{KTO}}\left(\omega \right)$ exhibits a THz intensity suppression very similar to $S_{\text{exp},\text{KTO}}\left(\omega \right)$ when compared to $S_{\text{exp},\text{ref}}\left(\omega \right)$, the TO1 dip strength is much weaker than that of $S_{\text{exp},\text{KTO}}\left(\omega \right)$. This suggests that the current ratio $\delta_{J}=\frac{J_{c,\text{samp}}\left(\omega \right)}{J_{c,\text{ref}}\left(\omega \right)}$ is not a constant, and $J_{c,\text{samp}}\left(\omega \right)$ should have a frequency structure associated with the TO1 mode as well.

The registration of the surface TO1 mode in $J_{c}\left(\omega \right)$ can be understood as a modulation of the transient current (*59*) due to electron-lattice interactions at the interface, as illustrated in Fig. 4A. Both elastic and inelastic scattering at the Py/KTO interface can modify the charge current. Because of the limited frequency range of $J_{c}\left(\omega \right)$ set by the ultrafast current surge and relaxation in the Py layer as seen in the Py/sapphire reference, only those inelastic processes within this THz frequency range can be registered in the detected signal, as the spintronic THz emission process filters out other higher-frequency components (>3 THz). For the present case of KTO or STO, the primary phonon involved in this process is the TO1 phonon. The conversion of the initial spin-polarized charge current into an in-plane charge current $J_{c}\left(\omega \right)$ plays an important role because the out-of-plane electron motion is converted to an in-plane one (*36*–*39*) allowing the electrons to couple to the transverse TO1 phonon (*59*).

To provide a quantitative description of this modulation of $J_{c}\left(\omega \right)$, we adopted an analytic model of the interaction of THz waves with a ferroelectric (*60*) that equally applies to the paraelectric case. We assume that the hot electron–TO1 coupling leads to an ionic displacement field *D*_TO1_, which produces a polarization modulation dP/dt at the KTO interface with *P* proportional to the dielectric function, ε(ω). The induced contribution (i.e., the polarization modulation) has a phase lag of $\frac{\pi }{2}+\phi$ to the driving current $J_{c}\left(t\right)$, where $\frac{\pi }{2}$ comes from the time derivative, and ϕ is the phase angle of the dielectric function (ε(ω) = $|\varepsilon \left(\omega \right)|e^{\mathrm{i\phi}}$). As ω sweeps through the TO1 mode frequency, this phase lag goes from $\frac{\pi }{2}$ to $\frac{3\pi }{2}$. At resonance, the phase lag is π, giving rise to destructive interference of this induced contribution with the driving current (Fig. 4C, inset). To illustrate this interference effect, if we model the time-dependent driving current as $J_{c}\left(t\right)\propto \text{cos}\left(\omega t\right)$, then the induced contribution (−dP/dt) is $\propto c_{\text{ind}}|\varepsilon |\omega \text{sin}\left(\omega t-\phi \right)$ with *c*_ind_ a microscopic constant proportional to *d*_P_/*c* with *d*_P_ the thickness of the polarization layer. Summing the two currents and Fourier transforming, this leads to an estimated current ratio $\delta_{J}^{\prime }\left(\omega \right)$ in the frequency domain: $\delta_{J}^{\prime }\left(\omega \right)={\left\{{\left[1-c_{\text{ind}}|\varepsilon |\omega \text{sin}\left(\phi \right)\right]}^{2}+{\left[c_{\text{ind}}|\varepsilon |\omega \text{cos}\left(\phi \right)\right]}^{2}\right\}}^{1/2}$. Using the model dielectric function (*58*), Fig. 4C shows the profile of $\delta_{J}^{\prime }\left(\omega \right)$ (the red curve) where the spectral dip arising from the surface TO1 mode is evident. Note that *c*_ind_ is found to be small, so the large contribution of −dP/dt to $\delta_{J}^{\prime }\left(\omega \right)$ is due to the largeness of ε.

We now estimate the THz spectrum by considering the contribution from both the dielectric response as well as the current source term, $S_{\mathrm{JZ},\text{KTO}}\left(\omega \right)=\delta_{J}^{\prime }\left(\omega \right)S_{Z,\text{KTO}}\left(\omega \right)$. As presented in Fig. 4B, $S_{\mathrm{JZ},\text{KTO}}\left(\omega \right)$ is in good agreement with the experimental data $S_{\text{exp},\text{KTO}}\left(\omega \right)$ in the spectral shape, intensity, and TO1 dip strength. At frequencies away from the TO1 mode, the contribution from Z(ω) predominantly suppresses the THz intensity compared to $S_{\text{exp},\text{ref}}\left(\omega \right)$. However, at the TO1 mode frequency $\omega_{\text{TO}1}$, the intensity suppression relative to $S_{\text{exp},\text{ref}}\left(\omega \right)$, i.e., $\frac{S_{\text{exp},\text{KTO}}\left(\omega =\omega_{\text{TO}1}\right)}{S_{\text{exp},\text{ref}}\left(\omega =\omega_{\text{TO}1}\right)},$ is about 10^−2^. This giant suppression is due in part to the contribution from the Z(ω), accounting for one order of magnitude $S_{Z,\text{KTO}}\left(\omega =\omega_{\text{TO}1}\right)/S_{\text{exp},\text{ref}}\left(\omega =\omega_{\text{TO}1}\right)$, with the remaining order of magnitude attributed to the $J_{c}\left(\omega \right)$ term. Because of the needed interaction of the spin-polarized electrons with the sample, the probe depth of this mechanism, i.e., the $J_{c}\left(\omega \right)$ term, is limited to the tunneling depth of the electrons. In the case of KTO or STO (*41*), this is about a few nanometers, which is consistent with the measurements (Fig. 1D).

The above discussion based on Eq. 1 is focused on Py/dielectric samples without a buffer layer inserted. Equation 1 was derived under the assumption that the metal layer can be treated as a perturbation and that the electric field is constant within the metal layer and so does not take into account any depth (*z*) dependence of the dielectric response due to the formation of a Schottky barrier at the interface. This approximation becomes even more questionable with the additional presence of a buffer layer. Ignoring any *z* dependence, the inclusion of the buffer layer would have two effects: (i) It could introduce another source (current) term to the numerator of Eq. 1; (ii) it adds an imaginary constant to the denominator of Eq. 1 that is equal to [*i*(ω/*c*) $\varepsilon_{B}$*d*] where ε_B_ is the dielectric constant of the buffer layer and *d* is the buffer thickness. This contribution from a buffer such as SiO_2_ or Al_2_O_3_ with a thickness of 100 nm is of order 10^−3^ in our THz window and so negligible. Thus, the dielectric response at the KTO/STO interface should still show up in the presence of such a buffer layer, which contradicts our observations (figs. S7 and S14). This would imply the necessity of a more complete treatment using the Poisson-Schroedinger equations to derive *E*(*z*), coupled with the wave equation for the THz radiation to calculate S(ω).

Our analysis based on Eq. 1 and our experimental results uncover the detection mechanism of SSTS. Although previous studies (*27*, *30*) have considered the effect of the sample’s dielectric response on the THz cavity radiation, this work unambiguously shown that both the cavity impedance term, Z(ω), and the source term, $J_{c}\left(\omega \right)$, contribute to detecting surface modes. Calculating the actual frequency profile of $J_{c}\left(\omega \right)$ as a result of the electron-lattice interaction would involve a detailed understanding of the interaction of the superdiffusive spin-polarized hot electrons in the Py with the phonons at the interface and presumably involves surface phonon dispersion and damping. Such complexities are beyond the present paper but would make an interesting topic for future study.

## DISCUSSION

We developed SSTS to enable experimental characterization of the sub-THz collective dynamics at surfaces that have not been achieved previously. This approach has revealed unique soft TO1 phonon dynamics at the surfaces of KTO and STO that deviate from the bulk, which is crucial for understanding interfacial superconductivity in quantum paraelectrics. In the context of quantum paraelectrics, our work provides an experimental platform to systematically study surface TO1 phonons that are hypothesized to be involved in the superconductivity of 2DEGs at their interfaces (*3*–*5*), for example in a superconductor heterostructure such as FM/AlO*_x_*/KTO with an AlO*_x_* layer (~ a few nanometers thick) so that SSTS can effectively probe the interfacial TO1 phonon modes (fig. S12). By gating this system, interfacial TO1 dynamics can be quantitatively measured as a function of the electric bias to explore the relationships between the TO1 phonon and interfacial superconductivity. Besides quantum paraelectrics, we envision our easy-to-use SSTS technique can be applied to a broad range of quantum materials for probing elusive collective excitations at THz frequencies.

## MATERIALS AND METHODS

### Sample preparation

Bare KTO and STO crystals, 500 μm thick, were obtained from MTI and Crystal GmbH, respectively, with KTO measuring 10 mm by 10 mm and STO 7.5 mm by 7.5 mm. In Py/KTO and Py/STO samples, a 3-nm Py layer was deposited on substrates at room temperature, using DC sputtering with 3 mtorr of Ar pressure and a rate of 0.7 Å/s. For Py/SiO_2_/KTO, a 100-nm SiO_2_ layer was radiofrequency sputtered at 5 mtorr before depositing 3-nm Py. For Py/BTO/KTO, BTO layers of varying thicknesses were deposited using pulsed laser deposition at 700°C and an oxygen pressure of 15 mtorr. The laser energy density was maintained at 2.6 J/cm^2^. After deposition, the samples were cooled to room temperature under the same pressure and transferred to the sputtering system. Then, 3-nm-thick Py was deposited on the BTO layer using the same dc sputtering method. For Py/Al_2_O_3_/STO, amorphous Al_2_O_3_ films were deposited on STO substrates via atomic layer deposition (ALD) using trimethylaluminum and water precursors in a Veeco Fiji ALD tool at 150°C under rough vacuum (~0.2 torr) with an Ar purge. A growth rate of 1.1 Å/cycle was confirmed by in situ spectroscopic ellipsometry on a Si wafer. Subsequently, 3 nm of Py was deposited on the Al_2_O_3_ layer using dc sputtering.

### THz generation and detection in SSTS

A schematic of the THz setup and measurements is provided in fig. S2. Samples were excited through the uncoated side by ~100-fs laser pulses with a wavelength of 800 nm (~1.55 eV) and a fluence of ~1.2 mJ/cm^2^. The emitted THz field induced birefringence in a <110>-cut 1-mm-thick ZnTe crystal, resulting in a rotation of the probe beam’s polarization. By using a λ/4 wave plate, a Wollaston prism, and a pair of balanced photodiodes, the THz waves were detected using an electro-optic sampling technique. The THz setup was operated in a nitrogen gas–purged environment to ensure 0% relative humidity. The detected THz field S(ω) is a convolution of the emitted THz field $E_{\text{near}}\left(\omega \right)$ and the response function of the detection scheme H(ω): $S\left(\omega \right)=H\left(\omega \right)E_{\text{near}}\left(\omega \right)$. Here, $H\left(\omega \right)=H_{P}\left(\omega \right)H_{d}\left(\omega \right)$, in which $H_{P}\left(\omega \right)$ accounts for the propagation of the THz pulse from the sample to the ZnTe crystal, $H_{d}\left(\omega \right)$ quantifies the electro-optic detection of the THz pulse by the probe beam in the ZnTe crystal. The response function H(ω) does not produce any pronounced spectroscopic peaks and dips within the THz detection window.

## Supplementary Materials

### This PDF file includes:

Figs. S1 to S14 References
