# Supplementary material for: Revealing subterahertz atomic vibrations in quantum paraelectrics by surface-sensitive spintronic terahertz spectroscopy
Source: Sci Adv. 2024 Nov 29;10(48):eads8601. doi: 10.1126/sciadv.ads8601 (PMC11606436; doi:10.1126/sciadv.ads8601)
Supplement: Supplementary file 1 — Figs. S1 to S14 References [file sciadv.ads8601_sm.pdf]

Science Advances
AAAS

Supplementary Materials for

Revealing subterahertz atomic vibrations in quantum paraelectrics by surface-sensitive spintronic terahertz spectroscopy

Zhaodong Chu *et al.*

Corresponding author: Michael R. Norman, norman@anl.gov; Anand Bhattacharya, anand@anl.gov; Haidan Wen, wen@anl.gov

Sci. Adv. **10**, eads8601 (2024)
DOI: 10.1126/sciadv.ads8601

This PDF file includes:

Figs. S1 to S14
References

Fig. S1. Cross-sectional TEM images of a Py/KTO (111) sample

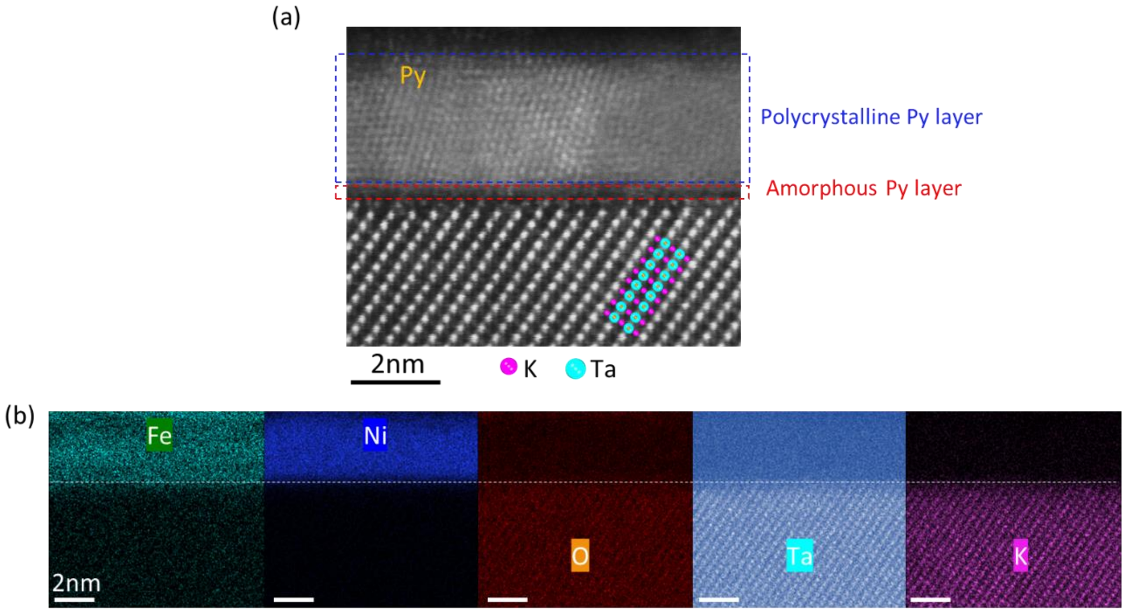

Fig. S1. (a) Cross-sectional high-angle annular dark-field (HAADF) scanning transmission electron microscopy (STEM) image of a Py/KTO (111) sample. (b) Elemental mapping images for the Py layer (Fe, Ni) and the KTO layer (K, Ta, O), illustrating the atomically precise interface between Py and KTO.

Fig. S2. Experimental Setup

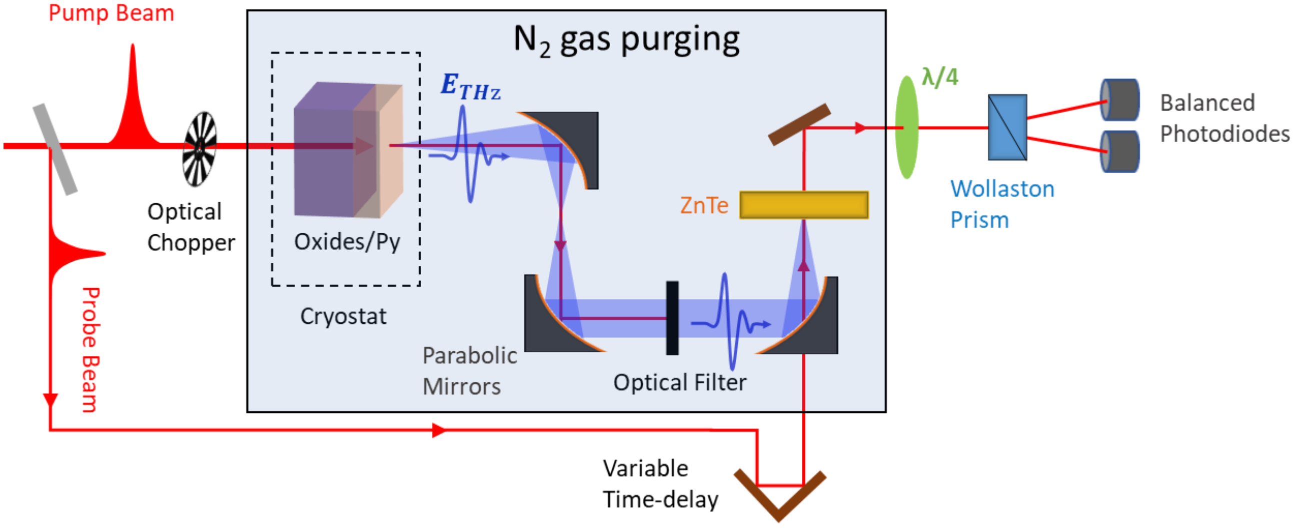

Fig. S2. A schematic of the THz spectroscopy setup.

Fig. S3. Comparison of THz emission from Py/STO, bare STO, and Pt/STO samples

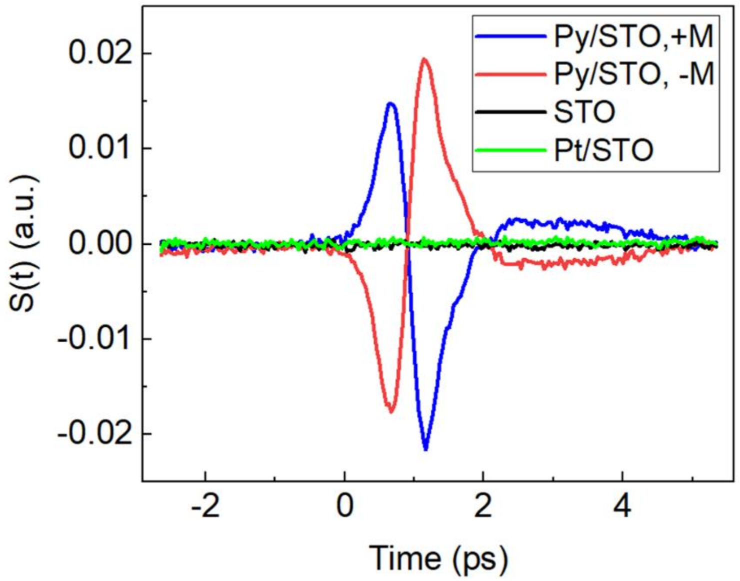

Fig. S3. Comparison of THz emission from Py/STO, bare STO, and Pt/STO samples. Pt: platinum. Here ±M labels the in-plane magnetization direction of the Py layer. Both Py and Pt are 3 nm thick. No THz signal is detected from the bare STO and Pt/STO samples, signifying the key role of spin-polarized electrons in the THz emission process.

Fig. S4. Reversing the THz polarization by changing the magnetization M and turning the sample around, respectively

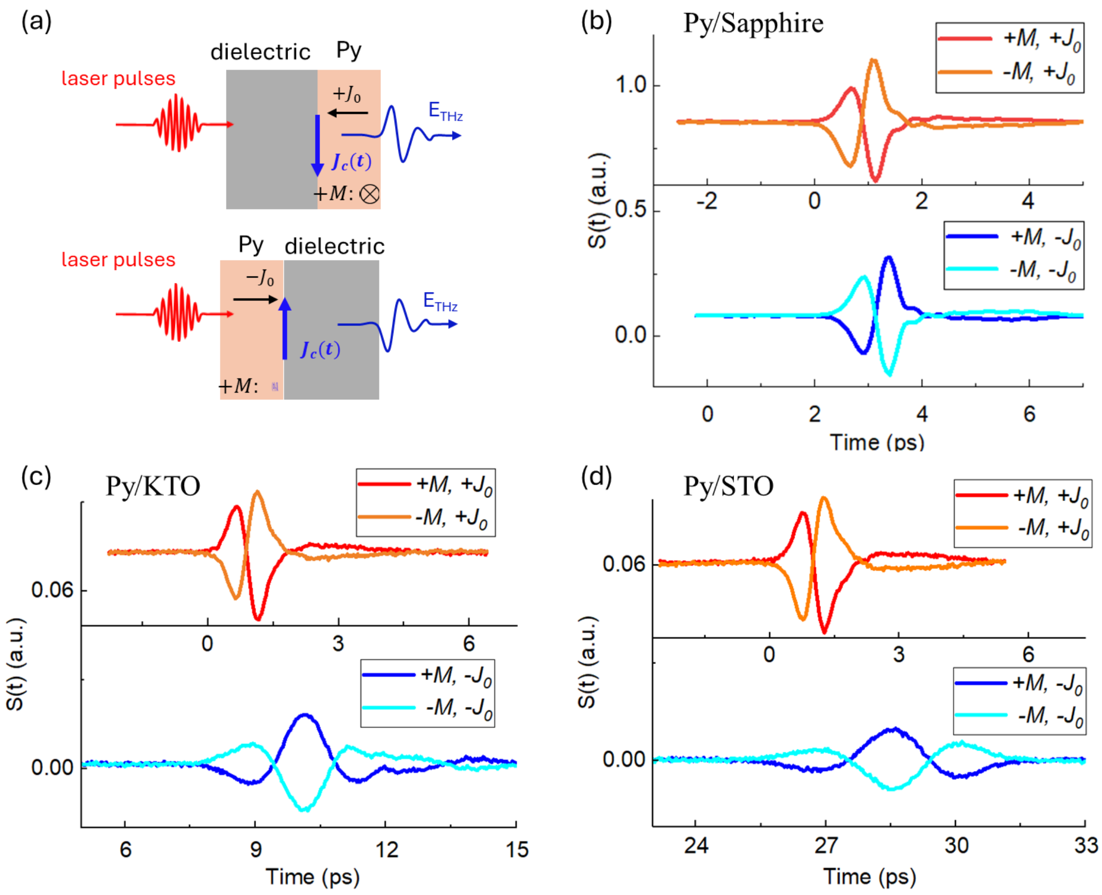

Fig. S4. (a) Schematic of THz emission from Py/dielectric samples under different pump-probe configurations. Top: Laser pumping from the dielectric side, with the spin diffusion current $\boldsymbol{J_0}$ (black arrow) denoted as $+J_0$, and the magnetization $\boldsymbol{M}$ direction (into the screen) denoted as $+M$. The interfacial transient current $\boldsymbol{J_c(t)} \propto \boldsymbol{J_0} \times \boldsymbol{M}$ is the source of THz radiation. Bottom: Laser pumping from the Py side by turning the sample 180^0 about $\boldsymbol{M}$, causing $\boldsymbol{J_0}, \boldsymbol{J_c}$ and the emitted THz to reverse their sign. The dielectric materials are sapphire (500 µm thick), KTO (200 µm thick) and STO (500 µm thick). (b-d) Time-domain THz waveforms at 295K, varying the magnetization directions (±M) and pump orientations ($\pm J_0$) in Py/sapphire (b), Py/KTO (c), and Py/STO (d). The reversal of the THz polarization in these samples upon rotating the sample (i.e., changing the sign of $\boldsymbol{J_0}$) confirms that the contribution from ultrafast demagnetization-induced magnetic dipole radiation is negligible in the measured THz field. Instead, spin-charge conversion (i.e., $\boldsymbol{J_c(t)} \propto \boldsymbol{J_0} \times \boldsymbol{M}$) through mechanisms such as the inverse spin Hall effect, anomalous Hall effect, skew scattering, and inverse Edelstein effect at the interface, dominate the THz emission. The time delays of the THz pulses between $+J_0$ and $-J_0$ cases arise from the refractive index mismatch of 800nm and THz lights within the dielectric materials. The THz profiles in Py/sapphire are nearly identical, differing only in sign, due to the negligible THz absorption by sapphire. In contrast, KTO and STO, with their larger refractive indices at THz frequencies, exhibit significant THz absorption in the "$-J_0$ " configuration, leading to differences in the THz time traces compared to the "$+J_0$" configuration.

Fig. S5. Temperature-dependent THz emission from a Py/sapphire sample

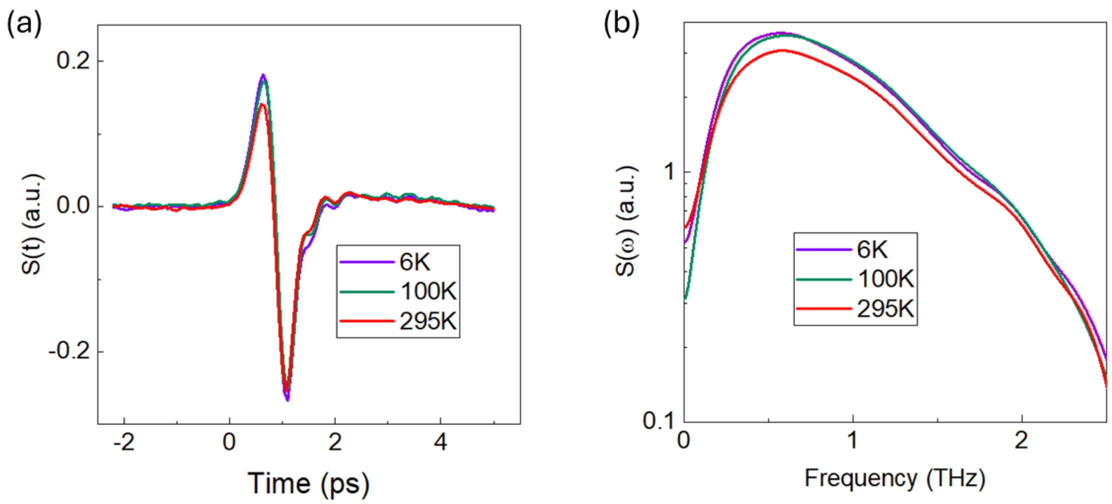

Fig. S5. Time-domain THz waveforms (a) and the frequency-domain THz intensity profiles (b) at 6K, 100K, and 295K. Minimal variations are observed between the THz signals at 6 K, 100K and 295 K, with no spectral dip features in the intensity profiles.

Fig. S6. THz reflection measurements

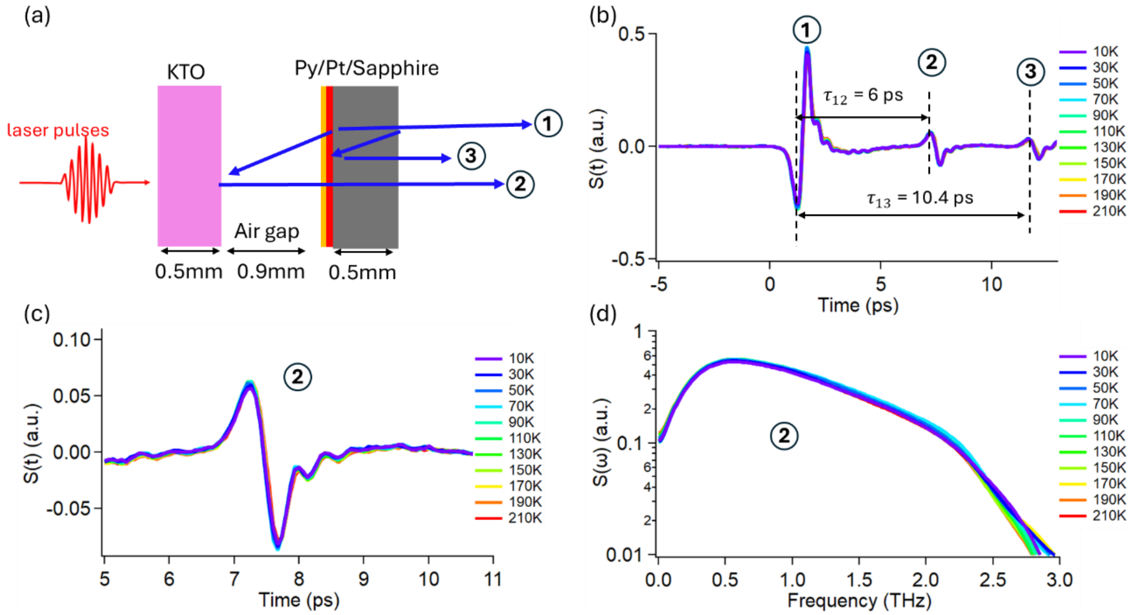

Fig. S6. Temperature-dependent THz reflection measurements. (a) Schematic of the THz reflection experiment setup. A Py/Pt/sapphire sample, commonly used as a spintronic THz emitter, serves as the THz source. It consists of a 3 nm thick Py layer, a 2 nm thick Pt layer, and a 0.5 mm thick (d_{sap}) sapphire substrate. The Py/Pt/sapphire sample is the THz source and placed close to a 0.5 mm thick KTO crystal with an air gap of d_{air}= 0.9 mm to separate the multi-reflected THz signals. The primary THz pulse emission is marked as ①, while ②, and ③ represent the subsequent reflected pulses from the interfaces/surfaces, as labeled in (a). Notably, ② is the reflection from the KTO surface. The time delays between these pulses and the primary pulse are: $\tau_{12} = n_{air} \cdot 2d_{air}/v_{THz} = 6$ ps, and $\tau_{13} = n_{sap} \cdot 2d_{sap}/v_{THz} = 10.4$ ps, where the refraction indexes of air and sapphire are n_{air} = 1 and n_{sap} ~ 3, respectively, d_{air} = 0.9 mm, d_{sap}= 0.5 mm, and v_{THz} = 3×10^8 m/s. (b) Time-domain THz profiles for pulses ① to ③ at various temperatures, showing little temperature dependence of the THz signals. (c) a zoom-in plot of the reflected pulse ②. (d) THz intensity profiles of the reflected pulse ② in the frequency domain, showing no spectral dip. These results demonstrate that the observed TO1 spectral dip in Fig. 1B is not due to the far-field absorption of the reflected THz wave.

Fig. S7: SSTS spectrum in the samples with THz-transparent spacers inserted

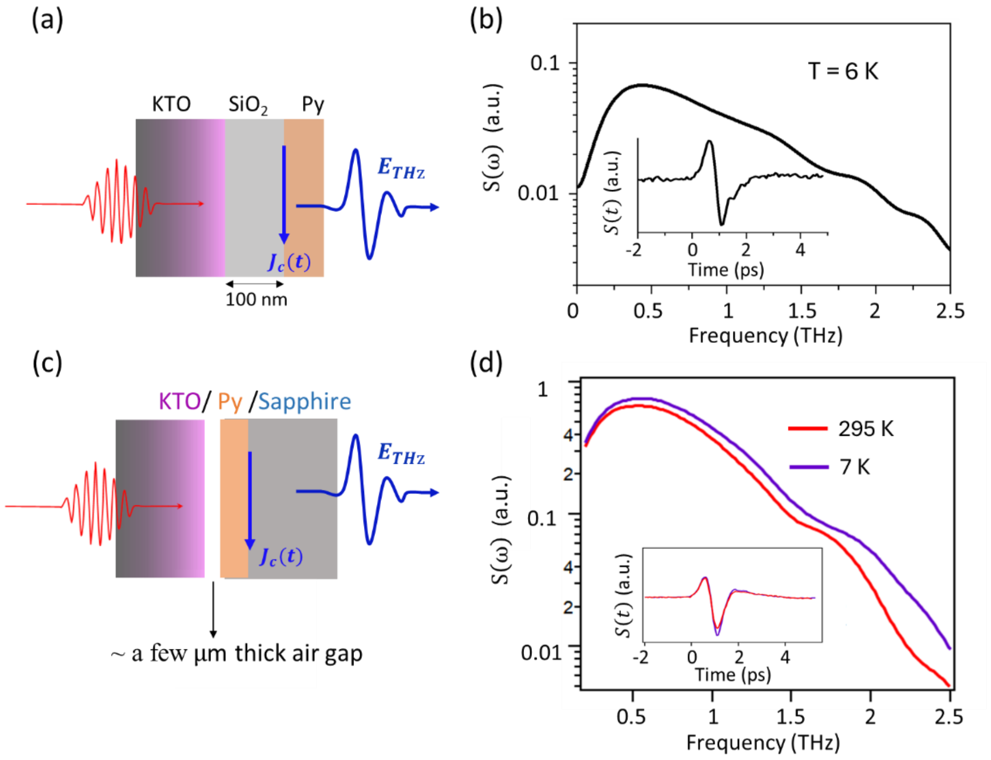

Fig. S7. THz emission from the Py/SiO_2/KTO (111) and KTO (111)/Py/sapphire samples. (a) shows a schematic of the SSTS measurements on the Py/100nm-thick-SiO_2/KTO (111) sample, where the THz field is detected at the Py side. (b) THz emission spectrum from the Py/100nm-SiO_2/KTO (111) sample at T = 6 K, where the TO1 mode is not detected. Inset: Time-domain waveform. (c) Schematic of the KTO (111) /Py/sapphire sample structure. a 3nm Py layer was deposited on one side of the sapphire substrate (500 µm thick). Subsequently, the KTO crystal was stacked on the Py-coated side, resulting in a KTO/Py/sapphire stacked configuration. This arrangement creates a few µm thick air gap between the Py and KTO surfaces. Such a gap separates the transient current $J_c(t)$ and the KTO surface. We illuminated the sample from the KTO side and detected the emitted THz field on the sapphire side. The backward-emitting component of the THz field traversed the vacuum gap, reached the KTO surface, and was then reflected towards the Py layer. The detected THz waves at temperatures of 7 K and 295 K are presented in (d), showing the minimal variation in the THz signal between 7 K and 295 K, and the absence of dip features in the THz intensity profiles. Our results show that the THz transparent spacer layer (e.g., 100 nm thick SiO_2 and a few µm thick air gap) blocks the detection of TO1 mode.

Fig. S8. Characterizations of Py/BTO/KTO samples

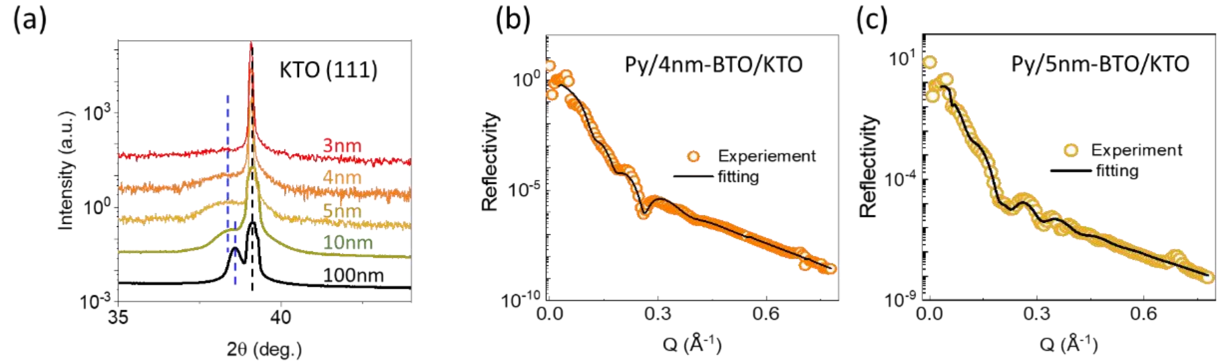

(d)

	Layers	Thickness (nm)	Roughness (nm)
Py/4nm-BTO/KTO	Py	3.1	1.3
	BTO	4.4	0.3
	KTO	500 µm	0.7
Py/5nm-BTO/KTO	Py	2.8	1
	BTO	5.7	0.2
	KTO	500 µm	0.5

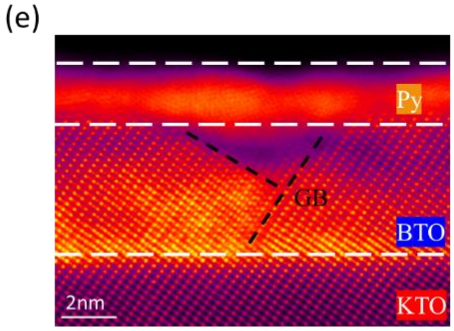

Fig. S8 (a) X-ray diffraction (XRD) data of Py/BTO/KTO (111) samples with varying BTO thicknesses (3 nm, 4 nm, 5 nm, 10 nm, and 100 nm). The X-ray energy is 8.04 keV. The 111 peak position of KTO is consistent with the measurements in Ref. 61. The 111 Bragg peaks of BTO, marked by blue dashed lines, are less prominent in thinner samples. The peak positions (2θ angle) for the 3-10 nm BTO films are ~ 0.2 degrees lower compared to the 100 nm BTO film, whose peak position is consistent with that of a non-strained BTO crystal[62,63]. (b) and (c) present the X-ray reflectivity (XRR) data for Py/4nm-BTO/KTO and Py/5nm-BTO/KTO samples, respectively. The solid lines represent the fitting curves, which were used to determine the thickness and roughness of each layer, as shown in (d). Note that the XRR intensity at low angles is slightly higher than 1 due to data correction with an error. (e) shows a cross-sectional HAADF STEM of the Py/4nm-BTO/KTO sample, where the grain boundaries (GB) in the BTO film are formed due to releasing the large lattice strain.

Fig. S9. Details of the FFT procedure and determining the error bar of the measured TO1 mode frequency

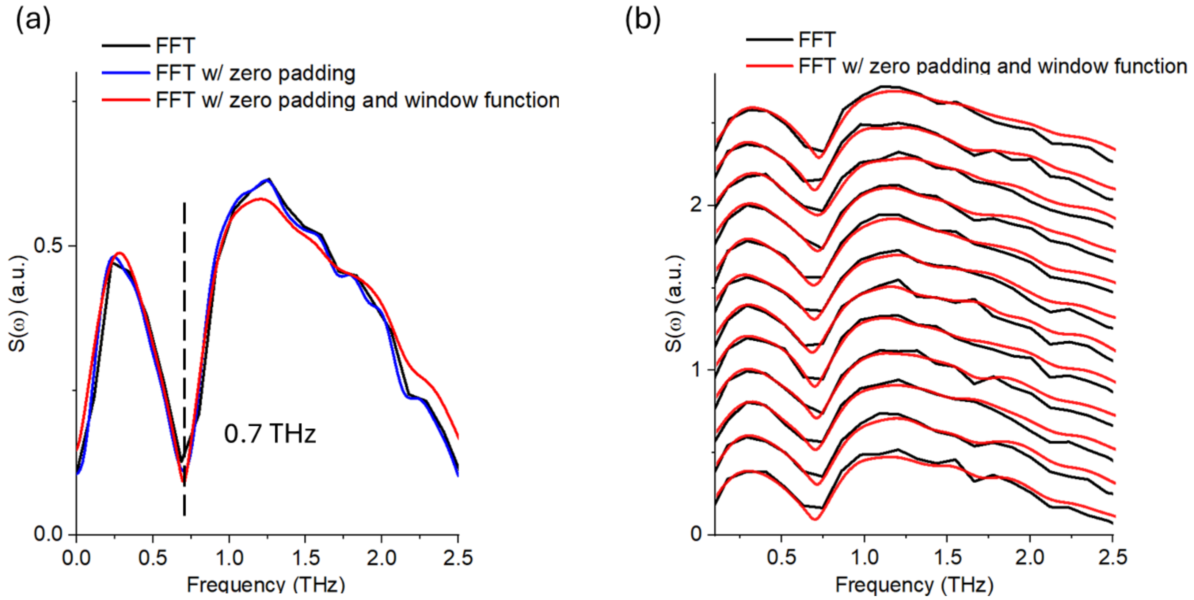

Fig. S9. Details of FFT procedure and estimation of the error bar for the measured TO1 mode frequency using data at 10K as an example. In this study, all THz time-domain trace data were averaged from more than ten individual repeated measurements to ensure consistency and a better signal-to-noise ratio. (a) Comparison of FFT results with and without zero padding and the Hanning window function. The black curve represents the FFT magnitude $S(\omega)$ of the averaged THz time-domain signal $S(t)$ from twelve repeated measurements, with a frequency step size of 0.11 THz. The blue curve shows the FFT with zero padding, which extends the time window for FFT, providing finer frequency resolution (~ 0.02 THz) without introducing artifacts. The red curve illustrates the FFT with both zero padding and the Hanning window function applied. These three FFT procedures yield very similar THz spectra in terms of shape and dip strength. For improving frequency resolution, all the frequency domain THz data presented in the main text and other figures in the Supplementary Materials were produced using both zero padding and the Hanning window function. (b) The THz spectra of the twelve individual repeated measurements. The black curves are the FFT magnitudes without zero padding and the Hanning window function. The red curves are the FFT with zero padding and the Hanning window function applied, from which, the TO1 mode frequency was determined as 0.7 ± 0.02 THz within a 99% confidence interval. The above analysis shows that we can determine the mode frequency with a higher frequency resolution in the FFT spectrum than the frequency interval determined by the length of the time delay window measured in the experiment.

Fig. S10: A complete set of temperature-dependent THz emission spectrum from a third Py/KTO (111) sample and a Py/STO(001) sample

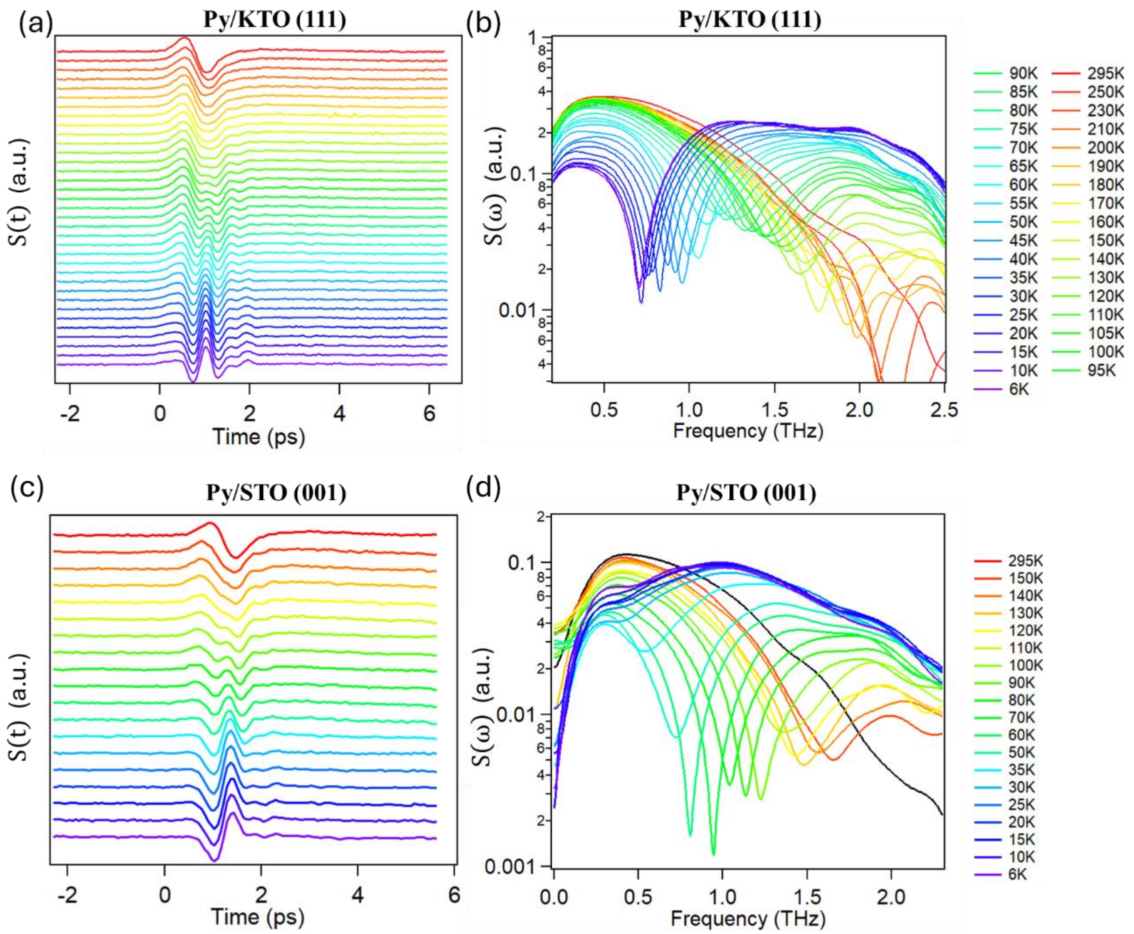

Fig. S10. Temperature-dependent THz field of another Py/KTO (111) sample represented in the time domain (a) and frequency domain (b). The thickness of this KTO crystal is 200 μm. The temperature-dependent surface TO1 mode in this sample agrees with that presented in the main text, where the KTO is 500 μm in thickness. The mode frequency levels off at ~ 0.7 THz when the temperature is below 15K. Temperature-dependent THz field from a Py/STO (001) sample depicted in the time domain (c) and frequency domain (d). The dips, corresponding to the surface TO1 mode, become broader, less pronounced, and imperceptible at temperatures below 35 K. Note that the spectral dip feature of the sub-meV mode is not obvious in this Py/STO (001) sample. The orientation dependence of this sub-meV mode needs further investigation, but is not unexpected given the known sensitivity of the antiferrodistortive transition in STO to surface orientation.

Fig. S11: Raman measurements of a Py/STO (001) sample

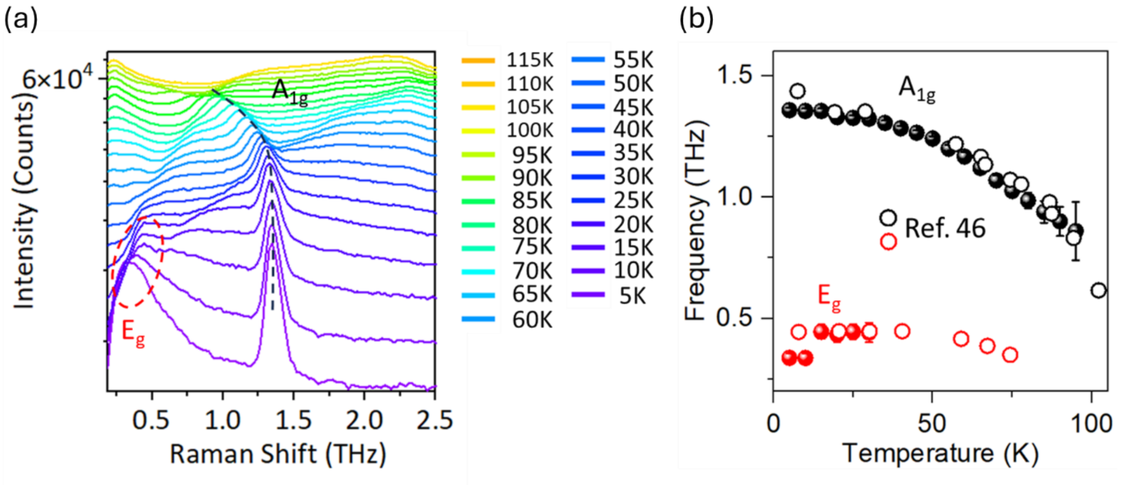

Fig. S11. (a) Temperature-dependent Raman scattering spectrum of a Py/STO (001) sample. Below the cubic to tetragonal phase transition temperature of 105 K in bulk STO, the soft R point phonon associated with it becomes mirrored at the Γ-point due to zone folding and splits into two Raman-active components of A_{1g} (marked by the black dashed curve) and E_g (highlighted by the red dashed circle) symmetries. (b) The temperature-dependent frequencies of A_{1g} and E_g in the Py/STO (001) sample, aligning with previous Raman measurements taken for bulk STO[46].

Fig. S12: THz emission spectra from Py/AlO$_x$/KTO (001) and Py/AlO$_x$/KTO (111) samples

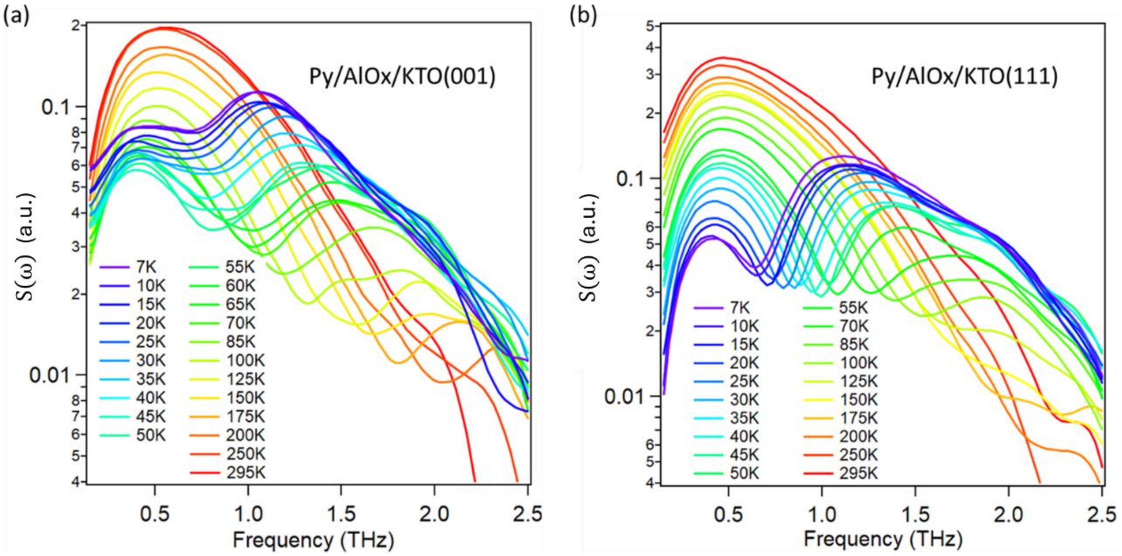

Fig. S12. Comparison of THz emission spectra from KTO 2DEG samples. The 2DEG is formed at the AlO$_x$(2 nm)/KTO interface due to oxygen vacancies. The 2DEG at the AlO$_x$/KTO (001) interface remains in the normal state down to 25 mK, and the 2DEG at the AlO$_x$/KTO (111) interface[3,4] becomes superconducting below ~ 2 K. Interestingly, at low temperatures, the spectral dips from the KTO TO1 mode at the (001) surface are much broader and shallower than those at the (111) surface.

Fig. S13. The contribution from the dielectric response in SSTS

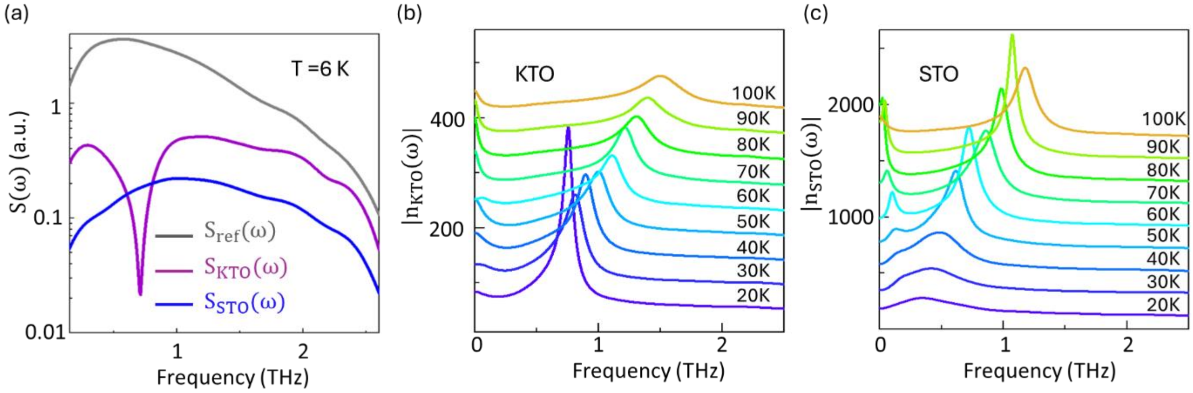

Fig. S13. (a) Comparison of the THz spectral intensity S(ω) of Py/KTO (purple) and Py/STO (blue) to a Py/sapphire reference (gray). The THz signal from KTO and STO is weaker because the refraction indices of KTO and STO are larger than that of sapphire. (b) and (c) show the calculated modulus of the refractive index of KTO surface ($n_{KTO}(\omega)$) and STO surface ($n_{STO}(\omega)$), respectively, by considering the contribution of the dielectric response alone. We estimate $Z_0 G = 1.7$ based on extrapolating the conductance of Py films to the thickness of 3 nm[64] and then exploit Eq. (2) to estimate $|n_{samp}(\omega)|$ (formally $|n_{samp}+Z_0G+1|$). These values assumed C=1 in Eq.(2) and would need to be multiplied by the current ratio (estimated to be C=1.62 from Fig. 4) for a better estimation. (b) illustrates the peak in the refraction index of KTO due to the TO1 mode that grows in strength and softens in energy as the temperature is lowered. (c) shows the refraction index of STO as a function of temperature. Similar to KTO, a TO1 mode is present and softens as the temperature decreases. But below about 100 K, a low-energy mode near 0.1 THz shows up that hardens as T decreases, while both this mode and the TO1 phonon broaden. Around 35 K, these two peaks merge into a single broad hump. This is consistent with the absence of the spectral dip at low T in Figs. 2D and 2F and implies an interaction between these two modes that is not evident in the bulk.

Fig. S14. THz emission with Al_2O_3 buffer layers

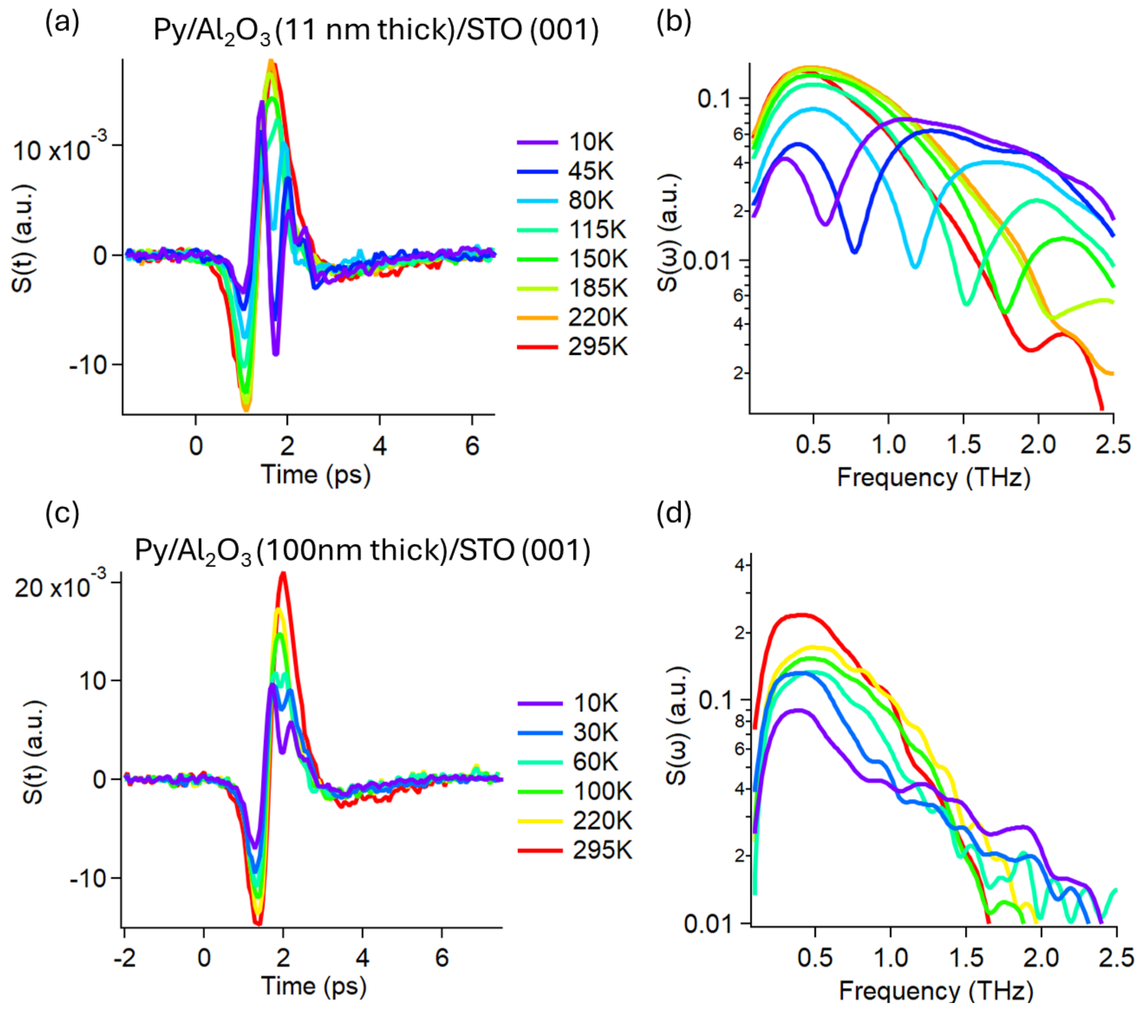

Fig. S14. THz emission in samples with a Al_2O_3 buffer layer. (a) and (b) THz emission spectrum of a Py/Al_2O_3 (11 nm thick)/STO (001) sample. The Al_2O_3 layer was deposited using atomic layer deposition (ALD). The transient current $J_c(t)$ is generated at the Py/Al_2O_3 interface, as the 11 nm-thick Al_2O_3 layer effectively blocks spin-polarized electron tunneling from the Py layer to the STO surface. The presence of the TO1 mode of STO should thus be attributed solely to the dielectric response, i.e. $Z(\omega)$. The dielectric constant of Al_2O_3 is much smaller than that of BTO[40], allowing the probe depth of near-field THz-matter interaction in Al_2O_3 to be deeper than that in BTO (Fig. 1D). (c) and (d) THz emission spectrum of a Py/Al_2O_3 (100 nm thick)/STO (001) sample. The Al_2O_3 layer in this sample was also deposited with ALD. Although slight changes in the time trace profiles were observed as the temperature decreased, the spectral dip is not prominent in the frequency domain. This suggests that a 100 nm Al_2O_3 layer deposited via ALD can block the detection of the TO1 mode for both $Z(\omega)$ and $J_c(\omega)$.

REFERENCES AND NOTES

1. A. D. Dunkelberger, C. T. Ellis, D. C. Ratchford, A. J. Giles, M. Kim, C. S. Kim, B. T. Spann, I. Vurgaftman, J. G. Tischler, J. P. Long, O. J. Glembocki, J. C. Owrutsky, J. D. Caldwell, Active tuning of surface phonon polariton resonances via carrier photo injection. *Nat. Photonics* **12**, 50–56 (2018).

2. S. Zhang, T. Wei, J. Guan, Q. Zhu, W. Qin, W. Wang, J. Zhang, E. W. Plummer, X. Zhu, Z. Zhang, J. Guo, Enhanced superconducting State in FeSe/$SrTiO_3$ by a dynamic interfacial polaron mechanism. *Phys. Rev. Lett.* **122**, 066802 (2019).

3. C. Liu, X. Yan, D. Jin, Y. Ma, H. W. Hsiao, Y. Lin, T. M. Bretz-Sullivan, X. Zhou, J. Pearson, B. Fisher, J. S. Jiang, W. Han, J. M. Zuo, J. Wen, D. D. Fong, J. Sun, H. Zhou, A. Bhattacharya, Two-dimensional superconductivity and anisotropic transport at $KTaO_3$ (111) interfaces. *Science* **371**, 716–721 (2021).

4. C. Liu, X. Zhou, D. Hong, B. Fisher, H. Zheng, J. Pearson, J. S. Jiang, D. Jin, M. R. Norman, A. Bhattacharya, Tunable superconductivity and its origin at $KTaO_3$ interfaces. *Nat. Commun.* **14**, 951 (2023).

5. Y. Yu, H. Y. Hwang, S. Raghu, S. B. Chung, Theory of superconductivity in doped quantum paraelectrics. *NPJ Quantum Mater.* **7**, 63 (2022).

6. C. Zhao, A. Zhu, S. Gao, L. Wang, X. Wan, A. Wang, W. H. Wang, T. Xue, S. Yang, D. Sun, W. Wang, Phonon resonance catalysis in NO oxidation on Mn-based mullite. *ACS Catal.* **12**, 12113–12122 (2022).

7. Y. Wu, J. Ordonez-Miranda, S. Gluchko, R. Anufriev, D. D. S. Meneses, L. D. Campo, S. Volz, M. Nomura, Enhanced thermal conduction by surface phonon-polaritons. *Sci. Adv.* **6**, eabb4461 (2020).

8. A. A. Sirenko, I. A. Akimov, J. R. Fox, A. M. Clark, H. Li, W. Si, X. X. Xi, Observation of the first-order raman scattering in $SrTiO_3$ thin films. *Phys. Rev. Lett.* **82**, 4500–4503 (1999).

9. V. N. Denisov, B. N. Mavrin, V. B. Podobedov, J. F. Scott, Hyper-Raman spectra and frequency dependence of soft mode damping in $SrTiO_3$. *J. Raman. Spectrosc.* **14**, 276–283 (1983).

10. J. Neu, C. A. Schmuttenmaer, Tutorial: An introduction to terahertz time domain spectroscopy (THz-TDS). *J. Appl. Phys.* **124**, 231101 (2018).

11. K. Moon, H. Park, J. Kim, Y. Do, S. Lee, G. Lee, H. Kang, H. Han, Subsurface nanoimaging by broadband terahertz pulse near-field microscopy. *Nano Lett.* **15**, 549–552 (2015).

12. H. T. Stinson, A. Sternbach, O. Najera, R. Jing, A. S. Mcleod, T. V. Slusar, A. Mueller, L. Anderegg, H. T. Kim, M. Rozenberg, D. N. Basov, Imaging the nanoscale phase separation in vanadium dioxide thin films at terahertz frequencies. *Nat. Commun.* **9**, 3604 (2018).

13. S. Berweger, C. C. Neacsu, Y. Mao, H. Zhou, S. S. Wong, M. B. Raschke, Optical nanocrystallography with tip-enhanced phonon Raman spectroscopy. *Nat. Nano.* **4**, 496–499 (2009).

14. C. Richter, H. Boschker, W. Dietsche, E. Fillis-Tsirakis, R. Jany, F. Loder, L. F. Kourkoutis, D. A. Muller, J. R. Kirtley, C. W. Schneider, J. Mannhar, Interface superconductor with gap behaviour like a high-temperature superconductor. *Nature* **502**, 528–531 (2013).

15. A. G. Swartz, H. Inouea, T. A. Merz, Y. Hikita, S. Raghu, T. P. Devereaux, S. Johnston, H. Y. Hwang, Polaronic behavior in a weak-coupling superconductor. *Proc. Natl. Acad. Sci. U.S.A.* **115**, 1475–1480 (2018).

16. S. Liu, A. Hammud, I. Hamada, M. Wolf, M. Müller, T. Kumagai, Nanoscale coherent phonon spectroscopy. *Sci. Adv.* **8**, eabq5682 (2022).

17. J. Langer, D. J. de Aberasturi, J. Aizpurua, R. A. Alvarez-Puebla, B. Auguié, J. J. Baumberg, G. C. Bazan, S. E. J. Bell, A. Boisen, A. G. Brolo, J. Choo, D. Cialla-May, V. Deckert, L. Fabris, K. Faulds, F. J. García de Abajo, R. Goodacre, D. Graham, A. J. Haes, C. L. Haynes, C. Huck, T. Itoh, M. Käll, J. Kneipp, N. A. Kotov, H. Kuang, E. C. Le Ru, H. K. Lee, J.-F. Li, X. Y. Ling, S. A. Maier, T. Mayerhöfer, M. Moskovits, K. Murakoshi, J.-M. Nam, S. Nie, Y. Ozaki, I. Pastoriza-Santos, J. Perez-Juste, J. Popp, A. Pucci, S. Reich, B. Ren, G. C. Schatz, T. Shegai, S.

Schlücker, L.-L. Tay, K George Thomas, Z.-Q. Tian, R. P. Van Duyne, T. Vo-Dinh, Y. Wang, K. A. Willets, C. Xu, H. Xu, Y. Xu, Y. S. Yamamoto, B. Zhao, L. M. Liz-Marzán, Present and future of surface-enhanced Raman scattering. *ACS Nano* **14**, 28–117 (2020).

18. H. Lourenço-Martins, M. Kociak, Vibrational surface electron-energy-loss spectroscopy probes confined surface-phonon modes. *Phys. Rev. X* **7**, 041059 (2017).

19. C. A. Gadre, X. Yan, Q. Song, J. Li, L. Gu, H. Huyan, T. Aoki, S. W. Lee, G. Chen, R. Wu, X. Pan, Nanoscale imaging of phonon dynamics by electron microscopy, *Nature* **606**, 292–297 (2022).

20. G. Benedek, J. P. Toennies, Helium atom scattering spectroscopy of surface phonons: Genesis and achievements. *Surf. Sci.* **299-300**, 587–611 (1994).

21. J. D. Axe, G. Shirane, Inelastic-neutron-scattering study of acoustic phonons in Nb_3Sn. *Phys. Rev. B* **8**, 1965–1977 (1973).

22. T. Dekorsy, H. Auer, C. Waschke, H. J. Bakker, H. G. Roskos, H. Kurz, V. Wagner, P. Grosse, Emission of submillimeter electromagnetic waves by coherent phonons. *Phys. Rev. Lett.* **74**, 738–741 (1995).

23. B. Guzelturk, M. Trigo, O. Delaire, D. A. Reis, A. M. Lindenberg, Dynamically tunable terahertz emission enabled by anomalous optical phonon responses in lead telluride. *ACS Photonics* **8**, 3633–3640 (2021).

24. X.-C. Zhang, B. B. Hu, J. T. Darrow, D. H. Auston, Generation of femtosecond electromagnetic pulses from semiconductor surfaces. *Appl. Phys. Lett.* **56**, 1011–1013 (1990).

25. Y. Huang, Z. Yao, C. He, L. Zhu, L. Zhang, J. Bai, X. Xu, Terahertz surface and interface emission spectroscopy for advanced materials. *J. Phys. Condens. Matter* **31**, 153001 (2019).

26. T. Kampfrath, M. Battiato, P. Maldonado, G. Eilers, J. Notzold, S. Mahrlein, V. Zbarsky, F. Freimuth, Y. Mokrousov, S. Blügel, M. Wolf, I. Radu, P. M. Oppeneer, M. Münzenberg, Terahertz spin current pulses controlled by magnetic heterostructures. *Nat. Nano.* **8**, 256–260

(2013).

27. T. Seifert, S. Jaiswal, U. Martens, J. Hannegan, L. Braun, P. Maldonado, F. Freimuth, A. Kronenberg, J. Henrizi, I. Radu, E. Beaurepaire, Y. Mokrousov, P. M. Oppeneer, M. Jourdan, G. Jakob, D. Turchinovich, L. M. Hayden, M. Wolf, M. Münzenberg, M. Kläui, T. Kampfrath, Efficient metallic spintronic emitters of ultrabroadband terahertz radiation. *Nat. Photonics* **10**, 483–488 (2016).

28. T. J. Huisman, R. V. Mikhaylovskiy, J. D. Costa, F. Freimuth, E. Paz, J. Ventura, P. P. Freitas, S. Blügel, Y. Mokrousov, Th. Rasing, A. V. Kimel, Femtosecond control of electric currents in metallic ferromagnetic heterostructures. *Nat. Nano.* **11**, 455–458 (2016).

29. C. Zhou, Y. P. Liu, Z. Wang, S. J. Ma, M. W. Jia, R. Q. Wu, L. Zhou, W. Zhang, M. K. Liu, Y. Z. Wu, J. Qi, Broadband terahertz generation via the interface inverse Rashba-Edelstein effect. *Phys. Rev. Lett.* **121**, 086801 (2018).

30. T. S. Seifert, L. Cheng, Z. Wei, T. Kampfrath, J. Qi, Spintronic sources of ultrashort terahertz electromagnetic pulses. *Appl. Phys. Lett.* **120**, 180401 (2022).

31. M. B. Jungfleisch, Q. Zhang, W. Zhang, J. E. Pearson, R. D. Schaller, H. Wen, A. Hoffmann, Control of terahertz emission by ultrafast spin-charge current conversion at Rashba interfaces. *Phys. Rev. Lett.* **120**, 207207 (2018).

32. Z. Feng, H. Qiu, D. Wang, C. Zhang, S. Sun, B. Jin, W. Tan, Spintronic terahertz emitter. *J. Appl. Phys.* **129**, 010901 (2021).

33. H. Qiu, L. Zhou, C. Zhang, J. Wu, Y. Tian, S. Cheng, S. Mi, H. Zhao, Q. Zhang, D. Wu, B. Jin, J. Chen, P. Wu, Ultrafast spin current generated from an antiferromagnet. *Nat. Phys.* **17**, 388–394 (2021).

34. E. Rongione, O. Gueckstock, M. Mattern, O. Gomonay, H. Meer, C. Schmitt, R. Ramos, T. Kikkawa, M. Mičica, E. Saitoh, J. Sinova, H. Jaffrès, J. Mangeney, S. T. B. Goennenwein, S. Geprägs, T. Kampfrath, M. Kläui, M. Bargheer, T. S. Seifert, S. Dhillon, R. Lebrun, Emission of coherent THz magnons in an antiferromagnetic insulator triggered by ultrafast spin–phonon

interactions. *Nat. Commun.* **14**, 1818 (2023).

35. A. El Hamdi, A. Levchuk, C. Gorini, M. Boselli, V. Juvé, T. O. Otomalo, G. Vaudel, S. Gariglio, P. Ruello, J-Y Chauleau, M. Viret, Conversion of angular momentum into charge at picosecond timescales in the $LaAlO_3/SrTiO_3$ interface. *Phys. Rev. B* **110**, 054412 (2024).

36. Q. Zhang, Z. Luo, H. Li, Y. Yang, X. Zhang, Y. Wu, Terahertz emission from anomalous Hall effect in a single-layer ferromagnet. *Phys. Rev. Appl.* **12**, 054027 (2019).

37. V. Mottamchetty, P. Rani, R. Brucas, A. Rydberg, P. Svedlindh, R. Gupta, Direct evidence of terahertz emission arising from anomalous Hall effect. *Sci. Rep.* **13**, 5988 (2023).

38. A. Comstock, M. Biliroglu, D. Seyitliyev, A. McConnell, E. Vetter, P. Reddy, R. Kirste, D. Szymanski, Z. Sitar, R. Collazo, K. Gundogdu, D. Sun, Spintronic terahertz emission in ultrawide bandgap semiconductor/ ferromagnet heterostructures. *Adv. Opt. Mater* **11**, 2201535 (2023).

39. B. F. Miao, S. Y. Huang, D. Qu, C. L. Chien, Inverse spin hall effect in a ferromagnetic metal. *Phys. Rev. Lett.* **111**, 066602 (2013).

40. M. Misra, K. Kotani, T. Kiwa, I. Kawayama, H. Murakami, M. Tonouchi, THz time domain spectroscopy of pulsed laser deposited $BaTiO_3$ thin films. *Appl. Surf. Sci.* **237**, 421–426 (2004).

41. K. Janicka, J. P. Velev, E. Y. Tsymbal, Quantum nature of two-dimensional electron gas confinement at $LaAlO_3/SrTiO_3$ interfaces. *Phys. Rev. Lett.* **102**, 106803 (2009).

42. Y. Su, J. Le, J. Ma, L. Cheng, Y. Wei, X. Zhai, C. Tian, Probing interface of perovskite oxide using surface-specific terahertz spectroscopy. *Ultrafast Sci.* **3**, 0042 (2023).

43. K. A. Müller, H. Burkard, $SrTiO_3$: An intrinsic quantum paraelectric below 4 K. *Phys. Rev. B* **19**, 3593–3602 (1979).

44. K. A. Müller, W. Berlinger, E. Tosatti, Indication for a novel phase in the quantum paraelectric regime of $SrTiO_3$. *Z. Phys. B Condens. Matter* **84**, 277–283 (1991).

45. H. Vogt, Refined treatment of the model of linearly coupled anharmonic oscillators and its application to the temperature dependence of the zone-center soft-mode frequencies of $KTaO_3$ and $SrTiO_3$. *Phys. Rev. B* **51**, 8046–8059 (1995).

46. P. A. Fleury, J. F. Scott, J. M. Worlock, Soft phonon modes and the 110°K phase transition in $SrTiO_3$. *Phys. Rev. Lett.* **21**, 16–19 (1968).

47. J. Petzelt, T. Ostapchuk, I. Gregora, I. Rychetský, S. Hoffmann-Eifert, A. V. Pronin, Y. Yuzyuk, B. P. Gorshunov, S. Kamba, V. Bovtun, J. Pokorný, M. Savinov, V. Porokhonskyy, D. Rafaja, P. Vaněk, A. Almeida, M. R. Chaves, A. A. Volkov, M. Dressel, R. Waser, Dielectric, infrared, and Raman response of undoped $SrTiO_3$ ceramics: Evidence of polar grain boundaries. *Phys. Rev. B* **64**, 184111 (2001).

48. Y. Ichikawa, M. Nagai, K. Tanaka, Direct observation of the soft-mode dispersion in the incipient ferroelectric $KTaO_3$. *Phys. Rev. B* **71**, 092106 (2005).

49. I. A. Akimov, A. A. Sirenko, A. M. Clark, J.-H. Hao, X. X. Xi, Electric-field-induced soft-mode hardening in $SrTiO_3$ films. *Phys. Rev. Lett.* **84**, 4625 (2000).

50. K. Inoue, N. Asai, T. Sameshima, Experimental study of the hyper-Raman scattering due to Raman inactive lattice vibration in $SrTiO_3$. *J. Physical Soc. Japan***50**, 1291–1300 (1981).

51. I. A. Akimov, C. Bernhard, A.Golnik, A. M. Clark, J. Hao, W. Si, X. X. Xi, Soft-mode hardening in $SrTiO_3$ thin films. *Nature* **404**, 373–376 (2000)

52. S. E. Rowley, L. J. Spalek, R. P. Smith, M. P. M. Dean, M. Itoh, J. F. Scott, G. G. Lonzarich, S. S. Saxena, Ferroelectric quantum criticality. *Nat. Phys.* **10**, 367–372 (2014).

53. K. Klyukin, V. Alexandrov, Effect of intrinsic point defects on ferroelectric polarization behavior of $SrTiO_3$. *Phys. Rev. B* **95**. 035301 (2017).

54. A. Stashans, A. S. Serrano, A quantum-chemical study of polar $SrTiO_3$ (110) surface and oxygen-vacancy defects therein. *Surf. Sci.*, **497**, 285–293 (2002).

55. R. Xu, J. Huang, E. S. Barnard, S. S. Hong, P. Singh, E. K. Wong, T. Jansen, V. Harbola, J. Xiao, B. Y. Wang, S. Crossley, D. Lu, S. Liu, H. Y. Hwang Strain-induced room-temperature ferroelectricity in $SrTiO_3$ membranes. *Nat. Commun.* **11**, 3141 (2020).

56. A. M. R. V. L. Monteiro, D. J. Groenendijk, I. Groen, J. de Bruijckere, R. Gaudenzi, H. S. J. van der Zant, A. D. Caviglia, Two-dimensional superconductivity at the (111)$LaAlO_3$/$SrTiO_3$ interface. *Phys. Rev. B* **96**, 020504(R) (2017).

57. A. G. Swartz, A. K. C. Cheung, H. Yoon, Z. Chen, Y. Hikita, S. Raghu, Harold Y. Hwang, Superconducting tunneling spectroscopy of spin-orbit coupling and orbital depairing in Nb:$SrTiO_3$. *Phys. Rev. Lett.* **121**, 167003 (2018).

58. S. Glinsek, D. Nuzhnyy, J. Petzelt, B. Malič, S. Kamba, V. Bovtun, M. Kempa, V. Skoromets, P. Kužel, I. Gregora, M. Kosec, Lattice dynamics and broad-band dielectric properties of the $KTaO_3$ ceramics. *J. Appl. Phys.* **111**, 104101 (2012).

59. P. Gu, M. Tani, "Terahertz radiation from semiconductor surfaces" in *Terahertz Optoelectronics*, Topics in Applied Physics, K. Sakai, Ed. (Springer, 2005), vol. **97**, pp. 63–98.

60. T. Chen, B. Wang, Y. Zhu, S. Zhuang, L.-Q. Chen, J. M. Hu. Analytical model and dynamical phase-field simulation of terahertz transmission across ferroelectrics. *Phys. Rev. B* **109**, 094305 (2024).

61. Y. Liu, Z. Liu, M. Zhang, Y. Sun, H. Tian, Y. Xie, Superconductivity in epitaxially grown $LaVO_3$/$KTaO_3$(111) heterostructures, *Chinese Phys. B* **32**, 037305 (2023).

62. R. Asiaie, W. Zhu, S. A. Akbar, P. K. Duttta, Characterization of submicron particles of tetragonal $BaTiO_3$. *Chem. Mater.* **8**, 226–234 (1996).

63. H. Hayashi, T. Nakamura, T. Ebina, In-situ Raman spectroscopy of $BaTiO_3$ particles for tetragonal-cubic transformation, *J. Phys. Chem. Solid* **74**, 957–962 (2013).

64. R. Rouzegar, L. Brandt, L. Nádvorník, D. A. Reiss, A. L. Chekhov, O. Gueckstock, C. In, M. Wolf, T. S. Seifert, P. W. Brouwer, G. Woltersdorf, T. Kampfrath, Laser-induced terahertz spin

transport in magnetic nanostructures arises from the same force as ultrafast demagnetization. *Phys. Rev. B* **106**, 144427 (2022).
